# An ecological approach to understanding the impact of sexual violence: a systematic meta-review

**DOI:** 10.3389/fpsyg.2023.1032408

**Published:** 2023-05-24

**Authors:** Dagmar Stockman, Laura Haney, Kasia Uzieblo, Heather Littleton, Ines Keygnaert, Gilbert Lemmens, Lesley Verhofstadt

**Affiliations:** ^1^Department of Experimental Clinical and Health Psychology, Faculty of Psychology and Educational Sciences, Ghent University, Ghent, Belgium; ^2^Department of Psychology, East Carolina University, Greenville, NC, United States; ^3^Department of Criminology, Vrije Universiteit Brussel, Brussel, Belgium; ^4^Forensic Care Specialists, Van der Hoeven Clinic, Utrecht, Netherlands; ^5^Lyda Hill Institute for Human Resilience, University of Colorado Colorado Springs, Colorado Springs, CO, United States; ^6^International Centre for Reproductive Health, Ghent University, Ghent, Belgium; ^7^Department of Head and Skin, Faculty of Medicine and Health Sciences, Ghent University, Ghent, Belgium

**Keywords:** sexual violence, social ecology, meta-review, psychotrauma, context, sexual violence impact

## Abstract

**Aim:**

A systematic meta-review was conducted to examine (1) the broad range of negative and positive individual and interpersonal changes following adult sexual violence, as well as (2) the risk/protective factors at multiple levels of the social ecology (e.g., individual, assault, and micro/meso/exo/macro/chronosystem factors)—influencing the impact of sexual violence.

**Methods:**

Searches of Web of Science, Pubmed, and ProQuest resulted in inclusion of 46 systematic reviews or meta-analyses. Review findings were extracted for summary and a deductive thematic analysis was conducted.

**Results:**

Experiencing sexual violence is associated with many negative individual and sexual difficulties as well as revictimization risk. Only a limited number of reviews reported on interpersonal and positive changes. Factors at multiple levels of the social ecology play a role in the intensity of these changes. Reviews including macrolevel factors were non-existent, however.

**Conclusion:**

Reviews on sexual violence are fragmented in nature. Although the use of an ecological approach is often lacking, adopting such a perspective in research is necessary for a fuller understanding of the multiple influences on survivor outcomes. Future research should evaluate the occurrence of social and positive changes following sexual violence, as well as the role of macrolevel factors in influencing post-assault outcomes.

## Introduction

According to the World Health Organization's definition, *sexual violence* is considered as “any sexual act against someone's will,^1^ committed by any person regardless of their relationship to the victim, in any setting” (World Health Organisation, 2015, p. 4). This definition of sexual violence can be applied to all genders and all ages and includes sexual harassment (i.e., non-contact sexual violence such as unwanted sexual comments), sexual assault (i.e., non-consensual bodily contact) and (attempted) rape [i.e., (attempted) non-consensual vaginal, oral or anal sex; Keygnaert, 2014]. Estimating the true prevalence of sexual violence is difficult due to the use of varying definitions of what sexual violence entails across studies. Not surprisingly, studies using more restrictive definitions (e.g., forcible rape) tend to find lower prevalence rates. International estimates of the prevalence of non-intimate partner sexual violence range from 3.3 to 21.0% of women over the age of 15 (Abrahams et al., 2014), and studies find that between 7.7 to 21.0% of women in United States have experienced sexual intimate partner violence (Bagwell-Gray et al., 2015). A European systematic review found prevalence estimates of adolescent/adult sexual violence ranging from 9.0 to 83.0% of women and 2.0 to 66.0% of men (Krahé et al., 2014).

Given that sexual violence seems to be a highly prevalent problem in society, it is vital to acknowledge the consequences of experiencing sexual violence in order to provide adequate support to survivors. In the past half century, the number of studies on the impact of sexual violence has increased tremendously, revealing a broad range of negative individual and social changes following sexual violence. In addition, research also supports that some survivors also report experiencing personal growth and other positive changes following violence. The severity of negative changes and the potential occurrence of positive changes following sexual violence depends on various factors (Hughes et al., 2005). Based on Bronfenbrenner's (1979, 1986) model, previous scholars have identified risk and protective factors for negative sexual violence outcomes at each level of the social ecology: individual level (e.g., sociodemographic variables, personality characteristics, coping skills, personal history), assault-related level (e.g., the relationship of the survivor with the offender, how coercion occurred, injury severity), micro-/mesosystem level (e.g., reactions from significant others toward disclosure), exosystem level (e.g., how institutions such as medical, legal and mental health systems provide support to the survivor), macrosystem level (e.g., [sub]cultural factors such as rape myth acceptance) and chronosystem level (e.g., lifespan transitions, prior victimization and revictimization; Neville and Heppner, 1999; Campbell et al., 2009).

Previous authors taking an ecological perspective toward understanding survivor adjustment and recovery following sexual violence have made important contributions to the literature by synthesizing the wealth of studies on this the topic (Neville and Heppner, 1999; Campbell et al., 2009). However, examination of this literature also highlights a number of gaps and limitations. For one, there are a limited number of systematic reviews of the research on the impact of sexual violence that includes ecological factors to understand how sexual violence affects survivors. Second, no prior reviews have simultaneously examined factors associated with negative individual (e.g., depression, posttraumatic stress disorder), interpersonal (e.g., changes in relationships with close and intimate others) and positive (e.g., posttraumatic growth, benefit finding) changes following adult sexual violence. Further, most existing reviews have not examined factors at multiple levels of the social ecology simultaneously, which is necessary to fully understand how these factors interact to affect survivor adjustment. A review which delineates factors associated with negative and positive individual and interpersonal changes following sexual violence is highly valuable for informing mental health policy, as well as for practitioners working with survivors (Hughes et al., 2005).

Given the large amount of available empirical evidence and research syntheses on the impact of sexual violence, and the broad range of variables related to our research topic, we conducted a “meta-review” (also referred to as “umbrella review” or “review of reviews”; Grant and Booth, 2009). Similar meta-reviews on the impact of child sexual abuse already exist (Maniglio, 2009; Hailes et al., 2019), but we are not aware of any published meta-reviews on the impact of adolescent and adult sexual violence (i.e., sexual violence that occurred when the survivor was 14 years old or older, hereafter referred to as “adult sexual violence” (ASA); Livingston et al., 2007). Thus, the overarching goal of the current study was to provide an overall picture of (1) the broad range of negative and positive individual and interpersonal changes following adult sexual violence, as well as (2) the risk/protective factors—including individual, assault, and micro/meso/exo/macro/chronosystem characteristics—influencing the impact of sexual violence, by summarizing the highest level of evidence (i.e., systematic reviews and meta-analyses).

## Methods

The current meta-review was conducted using a systematic and rigorous approach based on the guidelines of the Cochrane Collaboration (Higgins and Green, 2011). The meta-review protocol can be obtained upon request. The literature search was initially administered in November 2018 and then updated in March 2023.

### Literature search and inclusion criteria

A list of search terms was chosen after consulting review-experts and experts on the topic of sexual violence. Web of Science, PubMed and ProQuest were searched using the following search terms: Titles were searched using “(Sexual OR gender-based OR interpersonal) AND (exploitation OR trauma OR victimization OR violence OR coercion OR abuse OR intimidation OR force OR assault OR harassment) OR rape OR (forced OR coerced OR unwanted) AND (masturbation OR penetration OR intercourse OR sex)” whereas full texts were searched using “(Psychiatric OR Psycholog^*^ OR psychosocial OR social OR adaptation OR maladaptation OR adjustment OR maladjustment OR function^*^ OR dysfunction^*^ OR symptoms OR impact OR recovery OR sequelae OR aftermath OR consequences OR effect OR health OR growth OR well-being OR mental OR interpersonal OR risk OR protective OR moderat^*^ OR mediat^*^) and (Victim^*^ OR survivor^*^ OR family^*^ OR couple OR relationship OR friendship OR partner^*^).” Studies were retained if they were (a) systematic reviews or meta-analyses of the literature published in peer-reviewed scientific journals (i.e., no books, book chapters, book reviews, empirical studies, editorials or conference proceedings), (b) systematically reviewed studies on psychosocial changes after experiencing adult sexual violence or the factors influencing these changes, and (c) written in English. Reviews published until March 2023 were included. When possible, a filter for searching for reviews was added. Studies were excluded when they focused solely on child sexual abuse, non-sexual intimate partner violence or the physical sequelae of sexual violence, and when there was no focus on changes after sexual violence or factors influencing these changes (e.g., intervention, prevention and prevalence studies or studies focusing on perpetrators). References of selected papers were also screened to ensure all relevant studies for the current meta-review were included.

### Study selection

**Figure 1:**
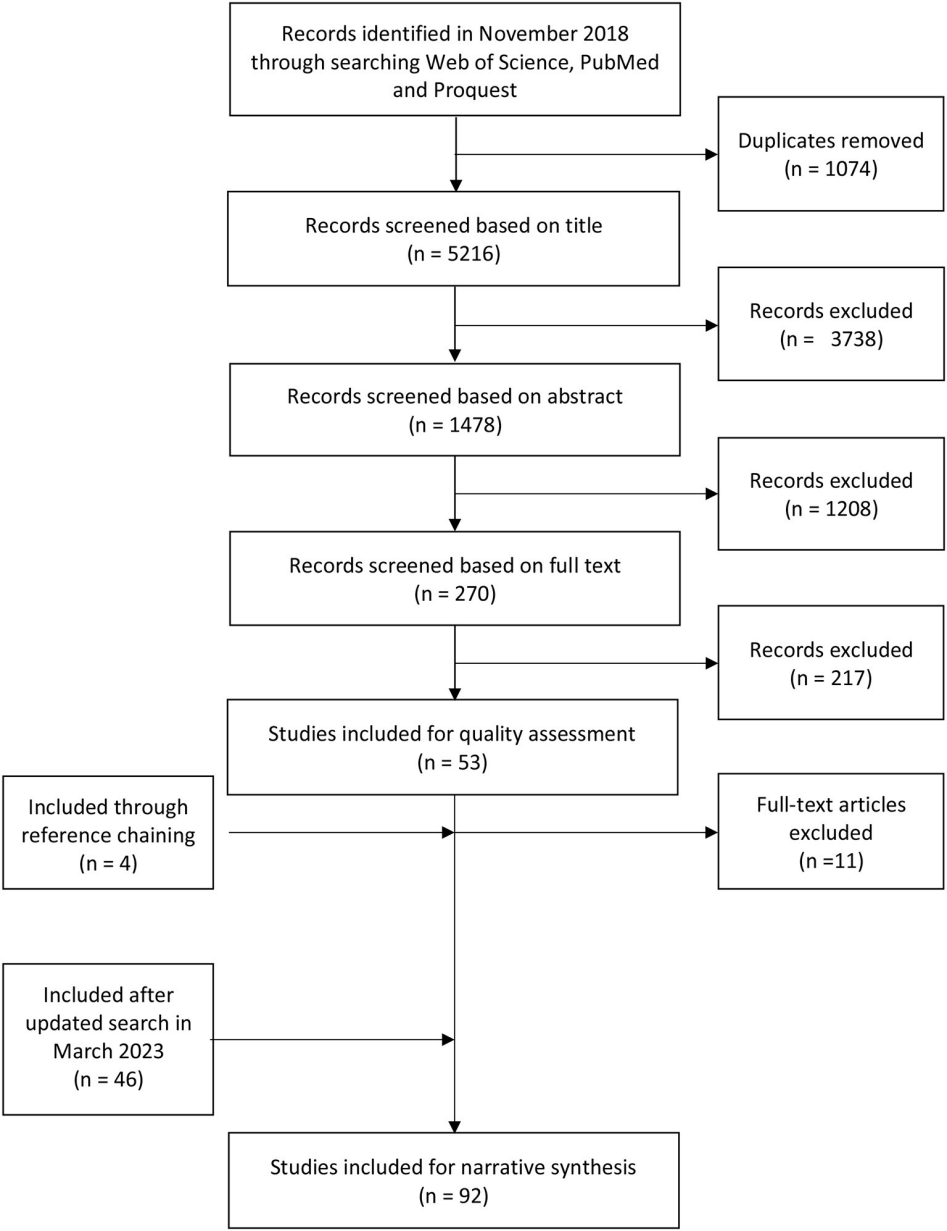
Selection of reviews.

After the initial search, 6,290 papers were identified, and after deleting duplicates 5,216 papers remained. The selection procedure comprised four phases (see Figure 1 for an overview of the selection procedure). In the first phase, the first and second author independently screened all titles for inclusion with an agreement of 86.1% (Kappa value = 0.79). Both authors discussed the disagreements until consensus was reached. In the second phase, both authors then screened the remaining 1,478 abstracts for inclusion resulting in an agreement of 85.2% (Kappa value = 0.80). Disagreements were discussed and resolved by both authors. In the third phase, the full texts of the remaining 270 papers were screened by the first author whereas the second author screened 50%, which resulted in an agreement of 72.1% (Kappa value = 0.54). Eligibility criteria on what is considered a systematic review and when to include reviews that do not exclusively report on adult sexual violence were re-evaluated. As such, reviews were included when they had conducted a systematic search and reported on studies systematically. Reviews that included at least one study on adult sexual violence/ sexual intimate partner violence were included as well. After this re-evaluation, papers without consensus were screened again, which increased the agreement to 82% (Kappa value = 0.70), resulting in 53 full texts. After screening the references of the selected papers, 4 studies were added, resulting in 57 papers selected for inclusion. In March 2023, an updated search resulted in the identification of 46 additional reviews. Altogether, this resulted in 99 reviews selected for inclusion.

### Quality assessment

In the fourth and final phase, the quality of the included studies was assessed by the first author. The second author assessed the quality of 50% of the reviews of the initial search. For quality assessment, the following seven criteria were administered (Bambra et al., 2009; Maniglio, 2009, 2013): the presence of (1) a well-defined research question, (2) a well-defined search strategy, (3) inclusion and exclusion criteria, (4) a thorough data extraction, (5) a quality assessment of the individual studies, (6) an appropriate synthesis, and (7) the inclusion of more than one researcher in the screening, selection, evaluation, and extraction processes. The agreement on the quality assessment of the 53 studies found after the initial search was 79.8% (Kappa value = 0.62). After evaluation, studies were categorized as being of high quality (a score of 6 or 7), fair quality (a score of 4 or 5) and poor quality (a score below 4). Of all included studies in this initial search, 45% (*n* = 26) were of high quality, 35% (*n* = 20) of fair quality and 20% (*n* = 11) of poor quality. Poor quality studies were excluded as they did not extract data from the individual studies in a detailed manner. The updated search yielded 33 (72%) high quality and 13 (28%) fair quality studies. Eventually, this resulted in a final number of 92 high and fair quality reviews for data extraction. An overview of the included (fair/high quality) reviews and their respective quality scores is shown in Supplementary Appendix A (accessible through https://osf.io/2fkm9/?view_only=6b48853fee7f4bf6ab9ec334bd369c13).

### Data extraction and coding

In a first coding phase, descriptive data on (1) paper characteristics (i.e., author, year of publication, journal), (2) search information (i.e., databases searched, whether a manual search was performed) and (3) study information (i.e., sexual violence definition, sample characteristics, changes assessed, risk and protective factors investigated) of the high and fair quality studies was extracted using an Excel spreadsheet (Maniglio, 2013). Reviews that also included primary studies outside the scope of this meta-reviews were included as well. In these cases, only the data relevant for the current meta-review was extracted (e.g., reviews on the impact of intimate partner violence were included if information on sexual intimate partner violence (SIPV) could be extracted). Descriptive information for each included article can be found in Supplementary Appendix A.

In a second phase, the data on the investigated changes and risk and protective factors were coded by the first author using deductive thematic analysis (Boyatzis, 1998; Braun and Clarke, 2006). More specifically, themes on changes and risk and protective factors were developed in advance based on empirical research on changes following sexual violence and Bronfenbrenner's (1979, 1986) model. Three themes related to changes following sexual violence were identified: (1) Negative individual changes, (2) Negative interpersonal changes and (3) Positive individual and interpersonal changes. Six themes related to risk and protective factors were identified: (1) Chronosystem factors, (2) Individual factors, (3) Assault-related factors, (4) Micro- and mesosystem factors, (5) Exosystem factors and, (6) Macrosystem factors. Subsequently, the results of all reviews were reread carefully, summarized comprehensively and categorized by the first author into the themes outlined above. Information within a theme that appeared to be on the same topic was then grouped together into a subtheme. When difficulties arose in the categorization of a specific outcome, risk or protective factor, the first and second author reflected upon the potential categorizations in order to reach consensus.

In the third and final phase, the themes and subthemes were described narratively together with their interrelations and summarized into tables. When available, effect sizes or other relevant statistical information were added (see Supplementary Appendices B, C—accessible through: https://osf.io/2fkm9/?view_only=6b48853fee7f4bf6ab9ec334bd369c13).

## Results

### Characteristics of included reviews

The 92 included review studies encompassed over 1,154 relevant primary empirical studies with over 11,807,073 respondents. The reviews were published between 1999 and 2023 and included primary studies published between 1980 and 2021. Thirty-one reviews had a quality score of 7, 28 reviews had a quality score of 6, 25 reviews had a quality score of 5 and eight reviews had a quality score of 4. Sixty-eight reviews only included relevant quantitative primary studies, three reviews only included relevant qualitative primary studies and one only included relevant mixed-method studies. In addition, 10 included quantitative and qualitative studies, nine included all three- quantitative, qualitative and mixed method studies and one review did not state whether included studies were qualitative or quantitative. Nine reviews were meta-analyses, eight reviews included both a systematic review and meta-analysis, and 75 were reviews that synthesized the data narratively including systematic reviews, critical reviews, rapid reviews, scoping reviews and meta-ethnographies. Three of the reviews included solely longitudinal studies.

### The impact of sexual violence

An overview of all documented positive and negative changes following sexual violence assessed in the included reviews can be found in Supplementary Appendix B (accessible through: https://osf.io/2fkm9/?view_only=6b48853fee7f4bf6ab9ec334bd369c13).

#### Negative individual changes

Seventy-four of the 92 included reviews investigated the association between sexual violence and negative individual changes.

##### Trauma- and stress-related disorders

Multiple reviews document a higher likelihood of receiving the diagnosis of *acute stress disorder (ASD), post-traumatic stress disorder (PTSD) or to report PTSD-related symptoms* (e.g., distress, arousal, avoidance of feared situations, intrusive thoughts, flashbacks, nightmares, other sleeping problems, anger and irritability and dissociative symptoms) among those who experienced sexual violence. Specifically, reviews show that a quarter to more than three-quarter of survivors report experiencing PTSD-related symptoms. Reviews including primary studies with control groups of non-survivors or survivors of other types of trauma report medium to large effect size differences in PTSD symptoms between groups (Van Berlo and Ensink, 2000; Classen et al., 2005; Wilness et al., 2007; Cook et al., 2011; Peterson et al., 2011; Steine et al., 2012; Trevillion et al., 2012; Wadsworth and Records, 2013; Ba and Bhopal, 2017; Chmielowska and Fuhr, 2017; Dworkin et al., 2017, 2023; Sparrow et al., 2017; Bows, 2018; Dworkin, 2018; Dame et al., 2020; Kahsay et al., 2020; Alessi et al., 2021; Forkus et al., 2021; Gallegos et al., 2021; Klein and Martin, 2021; Mazza et al., 2021; Zarchev et al., 2021; Klein et al., 2022; Lim et al., 2022; Spencer et al., 2023).

##### Anxiety disorders

In addition, sexual violence is associated with more *anxiety and specific fears* or phobias related to the sexual violence according to nine reviews (e.g., worrying it might happen again, fear of leaving the house, fear of sexually transmitted infections). More than one in 20 survivors to three-quarter of survivors report anxiety or anxiety-related behavioral changes, such as taking precautions to prevent experiencing another victimization, following the assault. Effect sizes comparing survivors to controls on anxiety symptoms range from small to large (Campbell et al., 2000; Ribeiro et al., 2009; Chen et al., 2010; Peterson et al., 2011; Wadsworth and Records, 2013; Larijani and Guggisberg, 2015; Ba and Bhopal, 2017; Chmielowska and Fuhr, 2017; Bows, 2018; Dworkin, 2018; Pulverman et al., 2019; Stewart et al., 2019; Dame et al., 2020; Kahsay et al., 2020; Pebole et al., 2021; Nicholas et al., 2022; Rani et al., 2022; Spencer et al., 2023). However, in some cases depression and anxiety are combined into one measure resulting in a combined effect size for both. In addition, two reviews reporting on anxiety found no significant differences when comparing sexual violence survivors to controls (Classen et al., 2005; Wadsworth and Records, 2013).

##### Mood disorders

Survivors are at higher risk for experiencing *mood disorders*. According to numerous reviews, up to two thirds of survivors report *depressive* symptoms and almost two in five patients with depression report having experienced ASA. In addition, when comparing survivors vs. controls and survivors of other trauma types, small to large effect sizes in depressive symptoms are found (Goodman et al., 1997; Campbell et al., 2000; Van Berlo and Ensink, 2000; Ullman, 2004; Classen et al., 2005; Ribeiro et al., 2009; Chen et al., 2010; Peterson et al., 2011; Beydoun et al., 2012; Dillon et al., 2013; Finneran and Stephenson, 2013; Mauritz et al., 2013; Wadsworth and Records, 2013; Alvarez-Segura et al., 2014; Ba and Bhopal, 2017; Chmielowska and Fuhr, 2017; Dworkin et al., 2017; Bows, 2018; Dworkin, 2018; Pulverman et al., 2019; Shamblaw et al., 2019; Dame et al., 2020; Godier-McBard and Jones, 2020; Kahsay et al., 2020; Paulson, 2020; Forkus et al., 2021; Klein and Martin, 2021; Mazza et al., 2021; Diez-Canseco et al., 2022; Klein et al., 2022; Nicholas et al., 2022; Rani et al., 2022; Lombardi et al., 2023; Spencer et al., 2023). In addition, 37% of patients with bipolar disorder report having experienced ASA, and a small to large effect size is found when comparing the bipolar disorder prevalence in survivors and controls (Mauritz et al., 2013; Dworkin et al., 2017). Finally, nearly two in five survivors report suicidal ideation and attempts, and medium to large effect sizes are found when comparing suicidality between survivors and controls (Ullman, 2004; Chen et al., 2010; Peterson et al., 2011; Dillon et al., 2013; Ba and Bhopal, 2017; Dworkin et al., 2017; Klein et al., 2022; Nicholas et al., 2022; Spencer et al., 2023). However, differences in depression and suicidal ideation between survivors and controls are not consistently confirmed in some reviews (Ullman, 2004; Ribeiro et al., 2009; Beydoun et al., 2012; Dillon et al., 2013; Finneran and Stephenson, 2013; Wadsworth and Records, 2013; Sparrow et al., 2017; Paulson, 2020; Forkus et al., 2021; Lim et al., 2022; Lombardi et al., 2023). Survivors also report feeling increased anger according to some reviews (Godier-McBard and Jones, 2020; Kahsay et al., 2020; Klein and Martin, 2021; Rani et al., 2022).

##### Substance use (disorders)

Having a sexual violence history is furthermore related to *substance use (disorders)*. Up to half of survivors report substance (ab)use and effect sizes for substance abuse range from small to medium when comparing survivors to controls and survivors of other trauma types (Goodman et al., 1997; Feldner et al., 2007; Meyer et al., 2011; Peterson et al., 2011; Finneran and Stephenson, 2013; Stockman et al., 2013; Wadsworth and Records, 2013; Heerde and Hemphill, 2016; Ba and Bhopal, 2017; Dworkin et al., 2017; Langdon et al., 2017; Sparrow et al., 2017; Dworkin, 2018; Stewart et al., 2019; Dame et al., 2020; Alessi et al., 2021; Forkus et al., 2021; Klein and Martin, 2021; Lim et al., 2022; Salim et al., 2022; Spencer et al., 2023). However, four reviews found no significant differences between ASA survivors and controls in substance use problems (Finneran and Stephenson, 2013; Heerde and Hemphill, 2016; Stewart et al., 2019; Forkus et al., 2021).

##### Other psychological disorders

Sexual violence is associated with other psychological disorders such as eating disorders, personality disorders and schizophrenic spectrum disorders. Specifically, nearly one in four *eating disorder* patients has experienced ASA, and more than half of survivors report disordered eating. Finally, multiple reviews found a small to medium effect size in disordered eating when comparing survivors to controls (Peterson et al., 2011; Bundock et al., 2013; Madowitz et al., 2015; Dworkin et al., 2017; Dworkin, 2018; Stewart et al., 2019; Dame et al., 2020; Klein and Martin, 2021; Spencer et al., 2023). In addition, between 39.0 and 87.0% of patients with a diagnosis of *borderline personality disorder* have experienced ASA (Peterson et al., 2011; Mauritz et al., 2013). One review, however, found no difference between ASA survivors and controls regarding the prevalence of borderline personality disorder (Trevillion et al., 2012). Finally, between six and 48.0% of patients with *schizophrenic spectrum disorders* report having experienced ASA (Goodman et al., 1997; Mauritz et al., 2013; Zarchev et al., 2021).

##### Self-perception

Six reviews found that survivors had a *less positive self-perception* (e.g., lower physical self-image, self-esteem, self-worth and self-respect) than healthy controls and survivors of other trauma types. In addition, 32.0% of survivors report negative self-perceptions (Campbell et al., 2000; Peterson et al., 2011; Finneran and Stephenson, 2013; Kouyoumdjian et al., 2013; Bows, 2018; Kahsay et al., 2020; Kouvelis and Kangas, 2021; Rani et al., 2022).

##### Health behavior

One review focused on how sexual violence is related to physical activity. Results supported individuals who regularly engaged in different forms of exercise differed in their likelihood of having a sexual violence history (with individuals who weight train having a higher prevalence of past sexual violence than runners) and that sexual violence survivors have an increased likelihood of having a sedentary lifestyle compared to controls (Pebole et al., 2021).

##### Academic and occupational functioning

Some reviews have focused on the impact of sexual violence on survivors' academic and occupational functioning. In general, up to a third of survivors report impairment in academic functioning (Peterson et al., 2011). Other reviews found that survivors report lower academic satisfaction, have lower GPAs, report lower academic self-efficacy and report increased academic distress, disengagement and absence (Stewart et al., 2019; Klein and Martin, 2021; Molstad et al., 2023). Sexual violence additionally is related to occupational functioning. Specifically, survivors report being more dissatisfied with their jobs, colleagues, and supervisors, as well as report decreased job productivity, commitment and attendance (Wilness et al., 2007; Stewart et al., 2019; Godier-McBard and Jones, 2020).

##### Somatic symptoms

Inconsistent results were found with respect to *somatic symptoms*. In reviews using survivor samples, up to 76.0% report suffering from somatic complaints including fatigue and chronic headaches (Ba and Bhopal, 2017; Kahsay et al., 2020). However, one review compared chronic headache sufferers and healthy controls and no differences were found with regard to ASA history (Paras et al., 2009). Two reviews similarly found no association of an ASA history with fibromyalgia (Romans and Cohen, 2008; Paras et al., 2009). When comparing survivors and controls, small to medium effect sizes were found for gastro-intestinal difficulties, psychogenic seizures, and chronic pelvic pain (Romans and Cohen, 2008; Paras et al., 2009; Wadsworth and Records, 2013).

##### Sexual changes

The association of sexual violence with *sexual changes*, is reported in various reviews that assessed the prevalence of sexual changes among SV survivor samples or compared samples of SV survivors and controls. Sexual violence is associated with high-risk sexual behavior with one review finding medium to large effect sizes for high-risk sexual behavior when comparing survivors to controls. Ten to nearly 90% of survivors report high-risk sexual behavior such as inconsistent condom use, using alcohol before sex, having casual sexual partners, or trading sex for money or drugs (Campbell et al., 2000; Maman et al., 2000; Messman-Moore and Long, 2003; Gielen et al., 2007; Meyer et al., 2011; Peterson et al., 2011; Finneran and Stephenson, 2013; Seth et al., 2013; Stockman et al., 2013; Wadsworth and Records, 2013; Bergmann and Stockman, 2015; Ba and Bhopal, 2017; Callan et al., 2021; Forkus et al., 2021; Lim et al., 2022; Spencer et al., 2023). In addition, reviews comparing survivors to controls found that survivors are more likely to practice non-monogamy and to have a greater lifetime number of sex partners, with one review finding small to medium-sized effects (Messman-Moore and Long, 2003; Gielen et al., 2007; Meyer et al., 2011; Finneran and Stephenson, 2013; Seth et al., 2013; Stockman et al., 2013; Forkus et al., 2021). In contrast, three reviews found no association of sexual violence history with decreased safe sex practices (Buller et al., 2014), trading sex for money or drugs (Finneran and Stephenson, 2013), non-monogamy (Wadsworth and Records, 2013) or lifetime number of sex partners (which also includes number of sex partners prior to sexual violence; Finneran and Stephenson, 2013).

Additionally, reviews report an association with a higher frequency of having sex (e.g., being six times more likely to have had sex more than 10 times in the past 3 months compared to controls; Maman et al., 2000; Messman-Moore and Long, 2003; Stewart et al., 2019), a lower sexual frequency and avoidance of sexual relationships with 30.0%−85.0% of survivors reporting aversion to and avoidance of sexual activity for at least 6 months (Van Berlo and Ensink, 2000; Madowitz et al., 2015; Stewart et al., 2019). No significant association of ASA with sexual frequency is reported in one review (Van Berlo and Ensink, 2000).

Alongside changes in sexual frequency, more than one in 10 to nearly six in 10 survivors report sexual difficulties (e.g., sexual dysfunction, decreased sexual satisfaction, decreased sexual desire, decreased sexual pleasure) with reviews finding small to medium-sized effects when comparing survivors, controls, and survivors of other trauma types (Campbell et al., 2000; Van Berlo and Ensink, 2000; Coker, 2007; Ba and Bhopal, 2017; Pulverman et al., 2019; Dame et al., 2020; Godier-McBard and Jones, 2020; Pulverman and Creech, 2021). However, two reviews found no difference between survivors and (military) controls regarding sexual wellbeing (Peterson et al., 2011; Pulverman et al., 2019). Two reviews mention the presence of sexual identity struggles among survivors (i.e., how an individual expresses his/herself sexually; for female survivors see Kouyoumdjian et al., 2013; for male survivors, see Peterson et al., 2011). Reviews also document that female survivors report powerlessness regarding engaging in sexual self-care, difficulties in rejecting unwanted sexual advances, and engaging in condom use negotiation, and decreased sexual assertiveness (Campbell et al., 2000; Maman et al., 2000; Meyer et al., 2011; Dillon et al., 2013; Kouyoumdjian et al., 2013; Stockman et al., 2013; Davis et al., 2023; Spencer et al., 2023).

##### (Sexual) revictimization

Sexual violence survivors also show an increased likelihood of becoming *revictimized*. Survivors vs. controls are 11 times more likely to experience intimate partner violence (Campbell et al., 2000; Maman et al., 2000; Meyer et al., 2011; Scoglio et al., 2021; Vitek and Yeater, 2021; Spencer et al., 2023). In addition, they have a greater likelihood of becoming sexually revictimized with a prevalence rate of 47% among adolescent survivors (Messman-Moore and Long, 2003; Classen et al., 2005; Walker et al., 2017). Taken together, overwhelming evidence supports that sexual violence is associated with risk for various types of individual psychopathology, including PTSD, anxiety, and affective disorders as well as academic and occupational impairment, elevated psychological distress, suicidality, substance use and sexual changes, including sexual risk behavior, sexual avoidance, and sexual dysfunction and revictimization.

#### Negative interpersonal changes

A total of 15 reviews investigate the association of sexual violence with negative interpersonal changes including three reporting on *general social changes*. As such, sexual violence is shown to be associated with social isolation (Kahsay et al., 2020; Rani et al., 2022; Davis et al., 2023). In addition, it was found that 10.0%−60.0% of survivors report social dysfunction (Ba and Bhopal, 2017) and that 66.0%−75.0% report distrust of others, 20.0% report withdrawal from social activities and 80.0% made social changes (e.g., joining a religious group, joining delinquent peer groups, avoiding areas; Peterson et al., 2011). In addition, SV survivors report experiencing interpersonal problems, including problems with peers and family (Messman-Moore and Long, 2003; Dillon et al., 2013; Godier-McBard and Jones, 2020; Kahsay et al., 2020; Spencer et al., 2023). Three reviews evaluated the impact of ASA on ongoing *intimate relationships. One* of these reviews focused on war-related sexual violence and found that a quarter of survivors report abandonment by their spouse (Ba and Bhopal, 2017). In addition, one review found that survivors report decreased relationship satisfaction and emotional intimacy compared to controls. Results on how sexual violence affects couple communication were inconsistent (Vitek and Yeater, 2021). Another review mentioned how survivors experienced a lower perceived control and increased feeling of powerlessness in their intimate relationships (Lim et al., 2022). An assault experience might also affect relationship initiation processes. Specifically, survivors report increased distrust in men and decreased dating behavior compared to controls (Stewart et al., 2019; Rani et al., 2022).

Three reviews suggest that having experienced sexual violence can also affect *parenting behavior* of both survivors' parents and survivors themselves. In the first review it was found that young adult survivors believe that their parents engaged in more restrictive parenting than before the disclosure (Halstead et al., 2017). The other two reviews found that survivors who are mothers terminate exclusive breastfeeding earlier compared to controls and have a lower likelihood of initiating breastfeeding (small to large effect sizes; de Souza Mezzavilla et al., 2018; Normann et al., 2020).

Altogether, the included reviews suggest that sexual violence is related to negative interpersonal changes within multiple domains and relationships. However, the evidence is limited.

#### Positive individual and interpersonal changes

Apart from the negative changes survivors encounter, four reviews suggest SV survivors may also report some positive changes or post-traumatic growth in the aftermath of sexual violence, as documented by four reviews (Ulloa et al., 2016; Elderton et al., 2017; Guggisberg et al., 2021; Klein and Martin, 2021). Fifty per cent of survivors report having changed a little to a great deal in the positive direction according to one review. Reported changes in this review included increased spirituality, a sense of wellbeing, improved relationships with their mothers, and more empathy toward others. However, when comparing SV survivors to individuals who lost a loved one in a motor vehicle accident, results show that SV survivors report less growth (Ulloa et al., 2016). In a second review, up to three quarter of survivors report growth in at least one of the following domains: *perception of self* (i.e., redefining themselves, more strength, recovery, enhanced self-efficacy, new possibilities), *perception of their relationship with others* (i.e., more closeness in relationships, receiving support from others, feelings of empathy toward others, engaging in social activism, helping others in similar situations), and their *outlook on life* (i.e., more appreciation of life, spiritual or religious changes, redefining priorities in life; Elderton et al., 2017). Similar themes were found in the review of Guggisberg et al. (2021).

In sum, alongside the negative individual and interpersonal changes survivors encounter after sexual violence, they might also experience changes in one or more so-called growth domains. However, one review documents that ASA may be less associated with positive changes than other forms of trauma.

### Risk and protective factors

An overview of the risk- and protective factors and the associations with negative and positive changes can be found in the Supplementary Appendix C (accessible through: https://osf.io/2fkm9/?view_only=6b48853fee7f4bf6ab9ec334bd369c13).

#### Chronosystem factors

Twenty review studies have identified chronosystem factors associated with ASA outcomes. One of these factors is the *time since the sexual violence* occurred. Specifically, reviews document that positive and negative individual changes (e.g., the initial development of and decrease in post-traumatic stress symptoms, sexual difficulties, growth and the likelihood of becoming revictimized) occur within the first months to the first year after a sexual violence incident (Van Berlo and Ensink, 2000; Classen et al., 2005; Cook et al., 2011; Ulloa et al., 2016; Elderton et al., 2017; Dworkin et al., 2023). Similarly, one review found that sexual trauma is usually not associated with delayed onset posttraumatic stress symptoms (Galatzer-Levy et al., 2018). One meta-analysis, however, found no association of time since the sexual violence with psychopathology (Dworkin et al., 2017).

The association between *having experienced childhood sexual trauma* before the ASA (vs. not having experienced childhood sexual trauma) and outcomes is inconsistent. Whereas in two reviews no significant association between having experienced childhood sexual trauma prior to the ASA and risk for reduced wellbeing was reported (Messman-Moore and Long, 2003; Classen et al., 2005), other reviews found a direct positive association of childhood sexual trauma with decreased wellbeing including lower post-traumatic growth, more depression, anxiety, PTSD symptoms, suicidality, and interpersonal and sexual difficulties. Further, an association between having a child sexual trauma history and individual risk factors for negative ASA outcomes was identified, including self-blame and less use of adaptive coping (Messman-Moore and Long, 2003; Ozer et al., 2003; Ullman, 2004; Classen et al., 2005; Wadsworth and Records, 2013; Hellman, 2014; Elderton et al., 2017; Kennedy and Prock, 2018; Salim et al., 2022; Molstad et al., 2023). Having experienced sexual violence more than once in adulthood is also associated with a greater likelihood of negative outcomes such as PTSD symptoms, depression, suicidality, sexual dysfunction, substance use, and lower GPA (Pulverman et al., 2019; Diez-Canseco et al., 2022; Rani et al., 2022).

A chronosystem factor that has received little attention, is *normative life events*. According to a review including qualitative research with survivors, one such event that has been found to influence the occurrence and severity of changes in female survivors was the process of giving birth, with some survivors reporting that this experience amplified feelings of shame and led to feelings of disconnection or alienation from their body (LoGiudice, 2016).

Taken together, chronosystem factors such as the time since the sexual violence may influence changes following sexual violence. The evidence for the influence of additional traumas or normative life events is either mixed or limited but seems to be consistent with work in the trauma field more broadly regarding the cumulative impact of multiple traumatic experiences.

#### Individual factors

In the next section, we will review individual factors identified in 32 reviews as being related to sexual violence sequelae, beginning with sociodemographic characteristics.

##### Sociodemographic characteristics

Sociodemographic characteristics identified in the included reviews are *gender, ethnicity, age, education level*, and *sexual identity*.^2^ The findings regarding *gender, ethnicity*, and *age* are rather contradictory. Some reviews suggest no gender differences with regard to a broad range of changes (Tolin and Foa, 2008; Dworkin et al., 2017; Bows, 2018). Other reviews, however, found gender differences including more PTSD symptoms, depression and anxiety symptoms, substance use, disordered eating, and high-risk sex among survivors who identified as women, and more distress, paranoia, hypomania, psychopathic traits, schizophrenia, sexual dysfunction, substance use (disorders), impairment in academic functioning, and suicidal behavior in survivors who identified as men (Peterson et al., 2011; Ba and Bhopal, 2017; Langdon et al., 2017; Godier-McBard and Jones, 2020; Forkus et al., 2021; Klein and Martin, 2021; Nicholas et al., 2022). Although not compared to women, men report concerns about their male gender role (i.e., behavior that is regarded as stereotypically male) after being victimized. In addition, hyper-heterosexual behavior and concerns about their sexual orientation are reported as well (Peterson et al., 2011; Godier-McBard and Jones, 2020).

The same pattern of inconsistency is found for ethnicity: a few reviews have found no differences among people who identify as White, Black/African American, Latine, and Native American with respect to overall psychopathology (Dworkin et al., 2017) and number of sex partners (Seth et al., 2013). Meanwhile, other reviews show that individuals who identify as White (vs. Black/African American) report more PTSD-symptoms (Wadsworth and Records, 2013) and that belonging to an ethnic minority group (vs. majority groups) is associated with more psychopathology (Hellman, 2014; Klein and Martin, 2021), but also with more post-traumatic growth (Ulloa et al., 2016; Elderton et al., 2017).

Differences among younger and older survivors are consistently reported in some reviews. However, the direction of these differences varied, with some reviews demonstrating increasing age to be a protective factor against negative changes (Ulloa et al., 2016; Dworkin et al., 2023) and a younger age to be a risk factor for negative changes (Hellman, 2014; Pulverman et al., 2019), and other reviews demonstrating the opposite pattern (Cook et al., 2011; Ulloa et al., 2016; Salim et al., 2022). Finally, three reviews show that holding a sexual minority identity (vs. heterosexual identity) is associated with greater psychopathology, and one review reported that having a *lower education level* relates to greater posttraumatic growth (Hellman, 2014; Ulloa et al., 2016; Klein et al., 2022; Salim et al., 2022).

##### Cognitive factors

Cognitive factors such as *self-blame* and *perceived control* are also associated with changes following sexual violence. Survivors who report more self-blame related to the sexual violence report more negative changes such as depression, PTSD symptoms and social withdrawal, and less post-traumatic growth compared to survivors who report less self-blame (Ullman, 1999; Van Berlo and Ensink, 2000; Hellman, 2014; Gong et al., 2019). In addition, it was found that survivors who experience more *perceived control* over their life and their recovery report more post-traumatic growth and less distress, compared to survivors who experience less perceived control (Ullman, 1999; Campbell et al., 2000; Hellman, 2014; Ulloa et al., 2016; Elderton et al., 2017).

##### Personality traits

There is some limited evidence that personality traits may play a role in the occurrence and intensity of certain changes following sexual violence. Three reviews on post-traumatic growth and resilience found that higher levels of traits such as hardiness and a greater ability to adapt to life changes (Ulloa et al., 2016; Elderton et al., 2017; Knight et al., 2022) combined with higher neuroticism levels are associated with more growth and less depression in survivors (Ulloa et al., 2016); whereas survivors who score higher on neuroticism, but not hardiness, report less growth (Ulloa et al., 2016). Additionally, having a sense of humor and having a positive outlook on life are shown to be associated with reporting finding meaning following the assault (Knight et al., 2022; Rani et al., 2022).

##### Coping behaviors

Multiple reviews highlight the importance of coping behaviors in the aftermath of the assault. Relying more on active coping strategies that involve working to solve the problems associated with the sexual violence or restructuring negative cognitions are associated with less distress and more post-traumatic growth (Ullman, 1999; Ulloa et al., 2016; Elderton et al., 2017; Pebole et al., 2021; Sinko et al., 2022), whereas relying more on avoidant coping strategies (i.e., coping strategies that serve to avoid emotions and cognitions related to the sexual violence) is associated with more distress, more high-risk sexual behavior and less post-traumatic growth (Ullman, 1999; Gielen et al., 2007; Hellman, 2014; Elderton et al., 2017; Kennedy and Prock, 2018). The effect of avoidant coping strategies is, however, not unequivocally negative as it depends on time since the sexual violence happened (as time passes avoidant coping becomes increasingly associated with more distress; Ullman, 1999).

##### Mental health

Multiple reviews show how pre-existing and post-assault mental health difficulties can affect other outcomes. For instance, post-traumatic stress and depression symptoms and broader psychological distress are associated with decreased sexual functioning, greater impairment in survivors' self-concept and difficulties in academic functioning (Pulverman et al., 2019; Bird et al., 2021; Klein and Martin, 2021; Kouvelis and Kangas, 2021). In addition, pre-existing alcohol abuse issues are associated with more PTSD symptoms 3 months following the assault, whereas alcohol use post-assault assault is associated with an increased likelihood of revictimization (Langdon et al., 2017; Gong et al., 2019).

In sum, the included reviews show that multiple individual factors are correlated with psychosocial changes following sexual violence. Although the evidence regarding most socio-demographics is inconclusive and the role of personality was not extensively investigated in the included reviews, many reviews have shown the importance of cognitive factors, coping, and mental health difficulties as risk or protective factors for negative and positive individual changes following sexual violence.

#### Assault-related factors

Assault-related risk and protective factors are documented in 12 reviews and include assault severity and perpetrator identity (gender and prior relationship of the survivor with the perpetrator).

Results among survivors show greater *assault severity* (e.g., completed rape vs. attempted rape, threat of using physical force, using physical force, being injured, actual life-threat, presence of a weapon, number of perpetrators) to be associated with more negative changes (Ullman, 2004; Classen et al., 2005; Feldner et al., 2007; Dworkin et al., 2017). However, results for sexual changes are inconsistent according to one review: penetrative sexual violence (vs. non-penetrative sexual violence) is associated with sexual difficulties, whereas the use of physical force (vs. no use of physical force) is not (Van Berlo and Ensink, 2000). With regard to positive changes, results are not unequivocal as one review reports a significant positive association between assault severity and positive changes (Ulloa et al., 2016) and another review documented no significant association between sexual assault severity and positive changes (Elderton et al., 2017).

Evidence regarding the role of *alcohol intoxication* during the assault is mixed according to one review. Both cross-sectional and longitudinal empirical studies found intoxicated survivors reported greater PTSD symptomatology, a longer recovery time, and greater substance use than survivors who were sober during the assault, whereas other studies found the opposite pattern or no significant differences between intoxicated and sober survivors (Gong et al., 2019).

Whether or not type of *prior relationship to the perpetrator* is a risk or protective factor for negative or positive changes following sexual violence is inconclusive. Four reviews suggest that having a prior (intimate) relationship to the perpetrator is not associated with negative or positive changes (Van Berlo and Ensink, 2000; Classen et al., 2005; Ulloa et al., 2016; Dworkin et al., 2017; Sparrow et al., 2017). However, in one review, sexual violence by a known (vs. unknown, identity not further specified) perpetrator was associated with sexual dysfunction, but not with changes in sexual frequency (Van Berlo and Ensink, 2000). One review found differential associations between sexual violence perpetrated by a current partner, past partner, or non-partner on PTSD-symptoms, stress, and dissociation. These patterns also differed among survivors who identified as White and survivors who identified as Black/African American, with non-partner sexual violence being unrelated to negative changes among White survivors and sexual violence by a past partner being unrelated to negative changes among Black survivors (Wadsworth and Records, 2013). For male survivors, there is some evidence that risk for negative outcomes may differ for those assaulted by male and female perpetrators. Specifically, those assaulted by male perpetrators may be more likely to experience distress, sexual identity confusion, and difficulties with trust as compared to those assaulted by female perpetrators (Peterson et al., 2011; Godier-McBard and Jones, 2020).

Overall, the reviews support that sexual assault severity is a risk factor for experiencing negative changes. How assault severity relates to positive changes and whether relationship with the perpetrator, the perpetrator's gender, and the survivors' substance use during the assault should be considered as a potential risk factor remains unclear.

#### Micro/meso system factors

Micro/meso system factors are identified in 17 reviews. The most extensively evaluated factor at the micro/meso system level is *social support*. Survivors who possess a supportive social network and are able to rely on this network report fewer negative changes post-assault (Ozer et al., 2003; Ullman, 2004; Wright et al., 2022). Separate from general social support, receiving more supportive reactions from others when disclosing the assault (e.g., listening, emotional support) is associated with more growth and resilience and less negative psychosocial changes, including PTSD and depression—either directly or indirectly through greater survivor reliance on adaptive coping strategies—than receiving fewer supportive reactions (Ullman, 1999; Van Berlo and Ensink, 2000; Hellman, 2014; Ulloa et al., 2016; Elderton et al., 2017; Halstead et al., 2017; Knight et al., 2022; Sinko et al., 2022). Receiving support appears to be especially important in the first 6 six months following the assault (Ullman, 1999). On the contrary, survivors who receive more unsupportive reactions when they disclose (e.g., disbelief or blame) report more negative psychosocial changes than survivors who receive fewer unsupportive reactions. This association is also mediated through greater survivor reliance on maladaptive coping strategies (Ullman, 1999; Hellman, 2014; Halstead et al., 2017; Kennedy and Prock, 2018; Gong et al., 2019; Salim et al., 2022; Wright et al., 2022). However, a recent meta-analysis showed that both supportive and unsupportive reactions are associated with greater psychopathology. According to that same meta-analysis, reactions that are generally considered as supportive in the literature have a differential effect compared to reactions that are actually *perceived* as being supportive by survivors. Specifically, perceived supportive reactions are found to be negatively associated—both cross-sectionally and prospectively—with psychopathology (Dworkin et al., 2019).

The *relationship of the survivor with the support provider* is differentially associated with psychosocial changes in response to disclosure of sexual violence. Although supportive reactions from family, friends and intimate partners following sexual violence disclosure are mostly perceived as helpful, they do not all relate to psychosocial adjustment. Receiving emotional support and other supportive reactions from friends is associated with better recovery than receiving emotional support from others (Ullman, 1999). Unsupportive reactions from family, friends and intimate partners are associated with negative psychosocial changes, of which unsupportive reactions from an intimate partner are associated with more negative psychosocial changes than unsupportive reactions from others (Ullman, 1999; Kennedy and Prock, 2018).

One review documented the *quality of the relationship* with family and one's intimate partner as a risk or protective factor for experiencing psychosocial changes following sexual violence. This review found that higher family closeness, defined as the emotional connectedness between family members (Manzi and Brambilla, 2014), is associated with decreased anxiety and depression symptoms 6 months after the sexual violence occurred, but not immediately after the event. In contrast, relationship quality with an intimate partner immediately after the assault is not associated with decreased anxiety and depression symptoms (Ullman, 1999).

Also, *living arrangements* (living alone, with partner or with family) and *relationship status* is a potential risk or protective factor. Survivors who were living with their family (vs. alone or with an intimate partner) at the time of the sexual violence report less PTSD-symptoms (Ullman, 1999). Marital status itself is not associated with negative individual changes (Ullman, 1999), but is negatively related to post-traumatic growth (Ulloa et al., 2016). Additionally, single survivors (vs. those in a relationship) report having more sex partners and more high-risk sex (Gielen et al., 2007; Seth et al., 2013).

Altogether, many reviews confirmed the importance of the absence and/or presence of (the perception of) support from close and intimate others as affecting psychosocial positive and negative changes following sexual violence.

#### Exosystem factors

Ten reviews report on the role of *formal support providers* such as law enforcement, medical personnel, and police personnel, in affecting survivor outcomes. Survivors who do not seek treatment report more suicidality (Peterson et al., 2011). Those who do seek treatment and those who receive supportive reactions from formal support sources experience fewer negative changes and more positive changes following sexual violence than survivors who receive fewer supportive reactions from formal support (Ullman, 1999; Ulloa et al., 2016; Elderton et al., 2017; Knight et al., 2022; Sinko et al., 2022; Wright et al., 2022). Conversely, unsupportive reactions from formal support providers are associated with negative psychosocial changes including PTSD and distress (Kennedy and Prock, 2018). Feeling pressured to reveal details about the assault to formal support providers is related to greater distress as well (Alessi et al., 2021). The potential effect of supportive reactions may differ depending on the source. More specifically, receiving more supportive responses from healthcare providers is strongly associated with psychosocial adjustment whereas responses from police were unrelated to adjustment (Ullman, 1999). Whether reactions that are perceived as being supportive by survivors have differential effects than those labeled as supportive in the literature remains unclear (Dworkin et al., 2019).

In sum, formal support providers' reactions are associated with both negative and positive changes survivors experience following sexual violence.

#### Macrosystem factors

Reviews explicitly including risk or protective macrosystem factors are nonexistent. Three reviews addressed the potential impact of certain macrosystem factors without including empirical studies on the association between macrolevel factors and change post-assault.

The potential impact of *gender norms* is mentioned in one review of outcomes among male survivors (Peterson et al., 2011). This review suggests that male survivors may sometimes worry about their sexual orientation and gender role reputation—due to societal male gender norms and ideas about masculinity—and therefore may underreport some negative changes (e.g., depression symptoms or decreases in sexual desire) while overreporting others (e.g., anger, alcohol use). Additionally, existing *rape myths* (i.e., stereotypical but false beliefs about sexual violence and survivors) in society are believed to affect survivors' cognitions and behaviors, as suggested by two reviews (Hellman, 2014; Kennedy and Prock, 2018). Although both reviews acknowledge the existence of rape myths and their potential impact on survivors, they do not include empirical research on the association between rape myths and changes following sexual violence.

Thus, reviews suggest that macrosystem factors may play a role in the impact of sexual violence, but empirical research on their impact is not available in the reviews.

## Discussion

The primary goal of this meta-review was to provide an ecological approach to understanding the impact of sexual violence by synthesizing the literature on (1) negative and positive individual and interpersonal changes that may occur following sexual violence, and (2) risk and protective factors at different ecological levels associated with these changes. Based on our thorough thematic analysis of the results of 92 systematic reviews, the following main conclusions can be drawn.

First, sexual violence is associated with many *negative individual and interpersonal changes*. Specifically, the association between sexual violence and trauma- and stress related disorders was investigated by a multitude of reviews and found to be consistent. Although not consistently established in all reviews, many found associations of sexual violence with risk for mood disorders, anxiety, substance use, suicidality, sexual dysfunction, and revictimization. Potential reasons for these inconsistencies may be the use of different sample types (e.g., clinical samples, community samples, no [appropriate] comparison groups) within and across review studies, different time frames during which outcomes were measured (e.g., some studies investigated outcomes immediately following the assault whereas in other studies there is an extended time between the assault and assessment of outcomes) and/or differences in how ASA is assessed across studies. Other potential forms of psychological distress and psychopathology following sexual violence are altered self-perceptions, disordered eating, personality disorders and—although not consistently found—somatic symptoms. A smaller proportion of reviews report on interpersonal changes, such as social isolation, distrust, interpersonal difficulties, and changes within relationships with significant others including friends, intimate partners, family, children, and parents, following sexual violence.

Second, alongside these negative changes, *positive changes* can occur following sexual violence. Although only a few reviews have investigated these positive changes, the results suggest that positive changes may occur in self-perceptions, the survivor's relationship with others and in the survivor's outlook on life. However, these positive changes may be less common following ASA as compared to other forms of trauma.

Third, experiencing changes following sexual violence is influenced by factors at multiple ecological levels. Many reviews document consistent significant associations between chronosystem (including time since the sexual violence), individual level (including self-blame, perceived control, and coping strategies), assault-related (including assault severity), micro/meso system (including [the absence] of a social network and (the perception of) (un)supportive reactions following disclosure), exosystem (including [un]supportive reactions from formal support providers) factors and changes following sexual violence. Macrosystem factors that are associated with changes following sexual violence remain unexplored in the included reviews, however.

Fourth, the current meta-review suggests that the aftermath of sexual violence involves a complex *interplay* of risk and protective factors in affecting survivor's recovery following sexual violence. For instance, the association between receipt of negative disclosure reactions and negative changes following sexual violence is (partially) mediated through self-blame and coping strategies. That is, receiving unsupportive reactions can lead to greater reliance on avoidant coping strategies and self-blame, exacerbating distress. The included reviews also document many associations between risk and protective factors in support of this interplay, but these associations fall outside the scope of this meta-review.

### Recommendations for practice

Findings of the current meta-review document the significant individual and interpersonal costs of sexual violence for the survivor. By adopting an ecological approach, the current study has identified a number of risk and protective factors documented in the research literature as relevant in understanding the impact of sexual violence. This evidence might be highly informative for practitioners working with survivors. For example, our findings support that a detailed assessment of the impact of sexual violence should not only focus on trauma and mood disorder (symptoms) but should also include a screening for sexual and interpersonal difficulties. In addition, repeated assessments of the impact of sexual violence are needed and should be scheduled throughout the 1-year time-window following sexual violence, as most psychosocial changes occur in the first months to the first year following sexual violence. Alongside the focus on potential changes following sexual violence, assessment efforts should also be directed at exploring risk and protective factors for survivors. For instance, assessing survivors' feelings of control, the extent to which they blame themselves, and the strategies they utilize to manage the assault, as well as the presence of an (un)supportive (in)formal network are vital in predicting survivors' (mal)adaptation. In the same way, assessment of how these support figures are dealing with the assault is important to identify whether survivors have interpersonal resources to assist them in their recovery. In line with this assessment, an intervention—tailored to the specific needs of each survivor—could be developed to reduce the impact of risk factors and to develop resources or to capitalize on existing ones. This intervention should aim to decrease negative individual and interpersonal changes following sexual violence and increase positive changes by, among other things, helping survivors regain a feeling of control over their life and their recovery, making them question their self-blaming cognitions and identifying and developing adaptive coping strategies. In addition, practitioners should support survivors' social network members in coping adaptively with the sexual violence and supporting the survivor. Finally, sexual violence awareness should be included in training of practitioners. A detailed assessment and intervention tailored to the survivors' needs can only take place when giving survivors the opportunity to talk about sexual violence and by asking whether they have had unwanted sexual experiences. In addition, it is vital to educate all types of formal support providers on how to react supportively to a sexual violence disclosure.

### Strengths and limitations

Conducting a meta-review allowed us to examine our broad research questions regarding the negative and positive individual and interpersonal changes following sexual violence, as well as the risk/protective factors associated with these changes (Pollock et al., 2020). Given the relevance of the multiple risk and protective factors as indicated by our review, adopting an ecological approach in research on sexual violence is necessary to advance our understanding. This meta-review is one of the few adopting such a perspective on the topic of sexual violence and thereby provides us with a broad overview of the literature. As such, it synthesizes many existing reviews and may aid in the development and adaptation of new and existing theories (Hunt et al., 2018).

However, this study also has its limitations. First, given the meta-review approach, detailed descriptions of the post-assault experiences of survivors is lacking. In addition, despite our focus on systematic reviews, this methodology runs the risk of not being exhaustive. Due to the immensely large number of fragmented studies, not every single study on this topic is documented in one of the included reviews. The list of potential changes and risk and protective factors described in the current review might, therefore, be incomplete. Moreover, most reviews include published studies only. Consequently, the current review may be biased as well. Further, our meta-review was based on a search of three major databases. Only including three databases might have limited the number of relevant reviews found. In addition, it is possible that our search terms were not broad enough to be able to find reviews that explicitly included research on the impact of macrolevel factors. Another limitation relates to the well-known problem of inconsistent definitions/assessments of sexual violence in research, leading to an inability to directly compare results across studies and reviews. Another limitation concerned challenges in categorizing some variables. As an example, while sexual identity was categorized as an individual factor, negative reactions from others toward sexual minority groups (microsystem factor) or discriminatory policies and practices toward sexual minority groups (macrosystem factor) are likely to affect the changes sexual minority survivors experience following sexual violence. Another typical difficulty in a meta-review is duplicate information because review studies on the same topic may include the same individual studies. Nevertheless, almost every single included review contributes to the current meta-review with unique empirical studies. By including all the published reviews, we are able to provide a more comprehensive snapshot of the literature than a single review which tends to have a narrower focus. In addition, by identifying areas that have received relatively little research attention, we are able to identify fruitful avenues for future research. The meta-review results also depend on the methods used and study quality of the individual reviews. It is only possible to include detailed and contextual study information in a meta-review based on the methods used to analyze empirical study findings in included reviews and how they present them (e.g., meta-analysis, narrative summary, providing an overview of all included empirical studies). When important information, such as effect sizes, is missing in individual reviews, overall statements on the impact of sexual violence can only be made with caution. A final limitation is that the current review cannot make strong claims about causality. Most empirical studies included in the reviews are non-experimental, cross-sectional or qualitative in nature. Moreover, many empirical studies on sexual violence may be biased as it is not known how survivors who choose to participate in research studies differ from survivors who choose not to participate.

### Suggestions for future research

The sometimes-inconsistent results regarding the changes following sexual violence suggest that more uniformity in sexual violence research, and specifically in reviews, is warranted. Reviews should clearly state what their definition of sexual violence is and how sexual violence was assessed in the included studies. In addition, more meta-analyses are necessary to review how sample types and time since the sexual violence affects outcomes and their relations with risk and protected factors. Furthermore, the current meta-review shows that not all existing reviews are of reasonable quality. Although the quality of more recent reviews has increased, future reviews should carefully follow review guidelines during the study selection and data extraction phase, including transparent communication on study selection and a rigorous extraction of all important methodological and statistical information on the included studies.

Of all changes and risk and protective factors included in the reviews, some received relatively little research attention. For instance, only a minority of the included review studies evaluated *interpersonal changes* following sexual violence. Every individual is imbedded in a social network to some extent which is of importance for our wellbeing, as is also illustrated by the impact of disclosure reactions on survivors' recovery. Consequently, quantitative and qualitative research on interpersonal changes and dynamics between survivors and individuals in their social network is warranted. The same is true for positive changes following sexual violence. Research on positive changes and their antecedents is important to understand survivors' recovery processes. Additionally, risk and protective macrosystem factors are not included in the existent review literature. This suggests that empirical studies on this topic are scarce. Accordingly, research on risk and protective macrosystem factors should be conducted. When we can identify if and how macrosystem factors play a role in survivors' recovery, this information can be used in both survivor intervention programs and efforts for creating societal changes with regard to how survivors are perceived. Finally, the included reviews demonstrate that the empirical research literature on sexual violence and its aftermath is still fragmented. However, the current meta-review supports that there is a complex interplay between factors at multiple levels of the social ecology in affecting adjustment among survivors. Therefore, taking an ecological perspective in future reviews and empirical studies is essential to delineate the multiple influences on sexual violence survivors' recovery.

## Conclusion

This meta-review compiles the evidence from existing reviews on the impact of sexual violence. It provides us with a comprehensive overview of the negative as well as potential positive individual and interpersonal changes that survivors of sexual violence experience, and the risk and protective factors for negative and positive changes following sexual violence. Our work sheds light on what is still unknown about risk and protective factors following sexual violence and may serve as a solid knowledgebase for researchers as well as clinicians working with survivors.

## Data availability statement

The raw data supporting the conclusions of this article will be made available by the authors, without undue reservation.

## Author contributions

LV, KU, and HL contributed to the initial idea for the study. DS developed search terms, carried out the search, created the database, conducted a thematic analysis, and wrote the first draft of the manuscript. DS and LH screened titles, abstracts and individual papers, and conducted a quality assessment on the individual papers. LH, HL, GL, KU, and LV revised the manuscript multiple times. All authors contributed to manuscript revision, read, and approved the submitted version.

## Footnotes

^1^Previous versions of this definition include “coercion”. Coercion implies overt and covert forms of verbal or physical persuasion or force. The sexual act is thus committed without a person's freely given consent. Free consent is not present when one is not able to give consent due to one's age, a disability, or lack of awareness, nor when one is not able to refuse freely [e.g., in the presence of weapons, due to (threats of) physical violence, intimidation, pressure or misuse of authority; Basile et al., 2014; World Health Organisation, 2019].

^2^Ethnicity and sexual identity were placed within the individual level as belonging to a certain minority group is an individual factor. Nevertheless, the authors acknowledge that the reason why ethnicity and sexual identity are risk factors are due to experiences with racism and/or discrimination (Wadsworth and Records, 2013; Ching et al., 2018). Experiences with racism and discrimination have as to our knowledge not yet been included in any reviews.
