# Supplementary material for: An ecological approach to understanding the impact of sexual violence: a systematic meta-review
Source: Front Psychol. 2023 May 24;14:1032408. doi: 10.3389/fpsyg.2023.1032408 (PMC10244654; doi:10.3389/fpsyg.2023.1032408)
Supplement: Supplementary file 1 [file Table_1.pdf]

APPENDIX A. Characteristics of included review studies

	Synthesis	Timeframe	Included studies	Region	Design	Sample	ASA context	Changes	R/P factor	Quality
**Alessi et al.(2021)**	Narr, Tab	2000-2020	Hopkinson et al., 2017<br>Kahn et al., 2018<br>Ogunbajo et al., 2018	United States, Canada	Quanti en quali	160 sexual and gender minority migrants from Europe, Africa, South-America, Central Asia, Middle East, age ns	Sexual violence during the migration process	I	E	6
**Alvarez-Segura et al. (2014)**	Narr, Tab	From 2011 onwards	Martin et al., 2006	United States	Quanti	95 women, age ns; community sample	SIPV	I		6
**Ba et al. (2017)**	Narr, Tab	1981-2014	***Johnson et al., 2010***<br>***Kelly et al., 2011***<br>Schalinski et al., 2011<br>Dossa et al., 2014<br>Hustache et al., 2009<br>Johnson et al., 2008<br>Kinyanda et al., 2012<br>Loncar et al., 2006	Democratic Republic of Congo, Liberia, Uganda, Croatia	Quanti and quali	5098 women and men, 14-83 years; clinical and community samples	War-based sexual violence	I, IP	I	7
**Bergmann et al. (2015)**	Narr, Tab	1997-2014	Fair et al., 2011<br>***Kalichman et al., 1998***	United States	Quanti, quali and mixed	273 women, age ns, clinical, community and student samples	SIPV	I		6
**Beydoun et al. (2012)**	Narr, Tab, Num, Graph	1980-2010	Bonomi et al., 2007<br>Coker et al., 2002<br>Vaeth et al., 2010<br>Yang et al., 2006	United States, Taiwan	Quanti	6052 women, 18-65 years, clinical samples	SIPV	I		5
**Bird et al. (2021)**	Narr, Tab	Until 2020	Andresen, 2019<br>***Blais et al., 2018***<br>Gobin, 2016<br>***Garneau-Fournier et al., 2018***<br>Pulverman, 2019<br>***Turchik et al., 2012***	Ns	Quanti	16363 female and male veterans, age ns	Military sexual violence		I	7
**Bows (2018)**	Narr, Tab	Ns	Ramsey-Klawsnik, 2003<br>Burgess, 2006<br>Jeary, 2005	United Kingdom, United States	Quanti, quali and mixed	>284 women and men, >60 years, clinical samples	Sexual elder abuse	I	I	5
**Buller et al. (2014)**	Narr, Tab, Num, Graph	Until 2013	Stephenson et al., 2010	United States	Quanti	665 MSM; age ns; community samples	SIPV	I		7
**Bundock et al. (2013)**	Narr, Tab	Until 2011	Leithner et al., 2009<br>Waller, 1991	Austria, United Kingdom	Quanti	491 Women, age ns, clinical	SIPV	I		7

APPENDIX A. Characteristics of included review studies (included)

	Synthesis	Timeframe	Included studies	Region	Design	Sample	ASA context	Changes	R/P factor	Quality
**Callan et al. (2021)**	Narr, Tab	1931-2019	Finneran et al., 2014<br>Stephenson et al.,2017	United States	Quanti	2650 gay and bisexual men, age ns, community camples	SIPV	I		7
**Campbell et al. (2000)**	Narr, Tab	Since 1995	***Campbell et al., 1999***<br>Champion et al., 1998<br>Davila et al., 1999<br>Holmes et al., 1998<br>Leenerts, 1999	United States (Survivors who identified as White, Black or Mexican).	Quanti and quali	604 women, age ns, clinical and community samples	SIPV	I	I	5
**Chen et al. (2010)**	Narr, Tab, Num, Graph	1980-2008	Chowdhary et al., 2008<br>Fergusson et al., 2002	New Zealand, India	Quanti	2548 women and men, 15-21 years, community samples	Any ASA	I		7
**Chmielowska et al. (2017)**	Narr, Tab	Until 2017	Robin et al., 1998	United States	Quanti	582 women and Men, >21 years, community samples	SIPV	I		6
**Classen et al. (2005)**	Narr, Tab	Ns	***Cloitre et al., 1997***<br>***Kalichman et al., 2001***<br>Krahe et al., 1999<br>***Paul et al., 2001***<br>***Wyatt et al., 1992***<br>Classen et al., 2001<br>***Cloitre et al., 2002***<br>Ellis et al., 1982<br>Field et al., 2001<br>***Frank et al., 1984***<br>Kellogg et al., 1997<br>Miller et al., 1978<br>Santiago et al., 1985<br>***Arata, 1999a***<br>Arata, 1999b<br>Arata, 1999c<br>Arata, 20000<br>***Gibson et al., 2001***<br>***Kessler et al., 1999***<br>***Proulx et al., 1995***<br>Roth et al., 1990<br>Collins, 1998<br>West et al., 2000<br>***Gidycz et al., 1993***<br>***Gydicz et al., 1995***<br>Humphrey et al., 2000<br>***Maker et al., 2001***<br>Banyard et al., 2001<br>***Cohen et al., 1987***<br>Gold et al., 1994<br>***Murphy et al., 1988***	United States	Quanti	14827 women and men, age ns, clinical and community samples	Any ASA	I	C, A	4

APPENDIX A. Characteristics of included review studies (included)

	Synthesis	Timeframe	Included studies	Region	Design	Sample	ASA context	Changes	R/P factor	Quality
			Ruch et al., 1991<br>Marhoefer-Dvorak et al.,1988<br>Sorenson et al., 1991<br>Koverola et al., 1996<br>Zlotnik et al., 1994<br>Rather et al., 1997<br>Wilson et al., 1999							
**Coker (2007)**	Narr, Tab	1966-2006	***Campbell et al., 1999***<br>Golding, 1996	United States	Quanti	3322 women, age ns, clinical and community samples	SIPV	I		6
**Cook et al. (2011)**	Narr	1980-2009	***Acierno et al., 2007***<br>***Atkeson et al., 1982***	Ns	Quanti	664 women, 15-87 years, clinical and community	Sexual elder abuse	I	C, I	6
**Dame et al. (2020)**	Narr, Tab	2009-2019	Aspin et al., 2009<br>Donne et al., 2018<br>Hequembourg et al., 2011<br>Sabido et al., 2015<br>Willis, 2009	United States, New Zealand, Brasil	Quanti, quali, mixed	4420 MSM and two-spirited people, 18-50 years old, community samples	Sexual violence outside of intimate relationship	I, IP		3
**Davis et al. (2023)**	Narr, Tab	Until 2022	Boadle et al., 2021<br>Levesque et al., 2021<br>Tarzia et al., 2020	Australia, New Zealand, Canada	Quanti en quali	399 women, 18-44 years, community samples	Stealthing following consensual sex	I		5
**de Souza Mezzavilla et al. (2018)**	Narr, Tab	Until 2017	Misch et al., 2014<br>Islam et al., 2017	Zambia, Ghana, Liberia, Tanzania, Zimbabwe, Kenya, Malawi, Nigeria, Bangladesh	Quanti	5248 mothers, 15-49 years, clinical samples	SIPV	IP		7
**Diez-Canseco et al. (2022)**	Narr, Tab, Num, Graph	From 2000 unwards	Abo et al., 2015<br>***Celik et al., 2007***<br>Dutra et al., 2010<br>Fang et al., 2018<br>Friborg et al., 2017<br>Gale et al., 2019<br>Gross et al., 2019<br>Hanson et al., 2015<br>Horn et al., 2017<br>Houle et al., 2011<br>Kim et al., 2021<br>Malik, 2021<br>Marsh et al., 2009	Egypt, Turkey, United States, China, Denmerk, Canada, South-Korea, Pakistan, Ethiopia, Norway, Spain	Quanti	622426 women and men from various professions, age ns	Sexual harrassment at the workplace	I	C	7

APPENDIX A. Characteristics of included review studies (included)

	Synthesis	Timeframe	Included studies	Region	Design	Sample	ASA context	Changes	R/P factor	Quality
			Mathisen et al., 2021<br>Matud et al., 2013<br>Milegan et al., 2016<br>Murdoch et al., 2007<br>***Mustaq et al., 2015***<br>Rugulies, 2020<br>Sumner et al., 2021<br>Wu, 2016<br>Yoo, 2008<br>Zhu et al., 2019							
**Dillon et al. (2013)**	Narr, Tab	2006-2012	Chen et al., 2009<br>Ellsberg et al., 2008<br>Ishida et al., 2010<br>***Naved et al., 2008***<br>Joseph et al., 2009	United States, Bangladesh, Paraguay	Quanti	Samples of women, sample size and age ns	SIPV	I, IP		4
**Dworkin (2018)**	Narr, Tab, Num, Graph	1970-2014	***Arata, 1999***<br>***Cheasty, 2002***<br>***Faravelli et al., 2004***<br>***Frank et al., 1987***<br>***Gutner et al., 2006***<br>Resick, 1991<br>***Halligan et al., 2003***<br>***Kaltman et al., 2005***<br>***McCutcheon et al., 2010***<br>***Testa et al., 2007***<br>Thompson et al., 2003<br>Wonderlich et al., 2001<br>Xu et al., 2013	United States, United Kingdom, Ireland	Quanti	33863 women and men, age ns, clinical, community and college samples	Any ASA	I		7
**Dworkin et al. (2017)**	Narr, Num, Graph	1970-2014	Abrams, 2008<br>***Acierno et al., 2007***<br>Amir, 1999<br>Aosved et al., 2011<br>***Arata, 1999***<br>Arata et al., 1996<br>Badour, 2011<br>Bailey et al., 1989<br>***Balsam et al., 2011***<br>Becker et al., 1984<br>Belik et al., 2009<br>***Ben-Ezra et al., 2010***<br>Betts et al., 2013<br>Binder-Brynes, 1991<br>Blanchard et al., 1996<br>Blix et al., 2011	Ns	Quanti	238623 women and men, age ns, clinical, community and student samples	Any ASA	I	C, I, A	7

APPENDIX A. Characteristics of included review studies (included)

	Synthesis	Timeframe	Included studies	Region	Design	Sample	ASA context	Changes	R/P factor	Quality
			Blix et al., 2012							
			Bolton et al., 2011							
			Borja et al., 2009							
			Boudreaux et al., 1998							
			Brener et al., 1999							
			Breslau et al., 1991							
			Brooks, 2007							
			Brown, 1996							
			Brown et al., 2003							
			Brownlie et al., 2007							
			Bryan et al., 2013							
			Burnam et al., 1988							
			Calhoun et al., 1982							
			Capitaine et al., 2011							
			Chang et al., 2003							
			Chang et al., 2014							
			Cheasty et al., 2002							
			Clark et al., 2012							
			Clements et al., 2009							
			Cohen et al., 2009							
			Collins et al., 2014							
			Conley et al., 2011							
			Cotton, 1980							
			Coxell et al., 1999							
			Cramer et al., 2012							
			Creamer et al., 2001							
			Cuevas et al., 2010							
			Danielson et al., 2009							
			Dansky et al., 1997							
			Davidson et al., 1996							
			Deliramich et al., 2008							
			DiVasto, 1985							
			Dorsett, 1995							
			Dubosc et al., 2012							
			Duke et al., 2008							
			Dworkin et al., 2014							
			Dworkin et al., 2016							
			Dyck, 1995							
			Eap, 2008							
			Edwards,1998							
			Eggers, 1985							
			Ehring et al., 2010							
			Elklit et al., 2014							
			Elliott et al., 2004							
			Ellis et al., 1981							
			Elwood, 2008							
			Evans-Campbell et al., 2006							

APPENDIX A. Characteristics of included review studies (included)

	Synthesis	Timeframe	Included studies	Region	Design	Sample	ASA context	Changes	R/P factor	Quality
			Fanslow et al., 2004							
			Faravelli et al., 2004							
			Filipas et al., 2006							
			Forman-Hoffman et al.,2012							
			Frank et al., 1987							
			Frazier et al., 1994.							
			Frazier et al., 2009							
			Frazier et al., 2004							
			Freedy et al., 2010a							
			Freedy et al., 2010b							
			Fusé, 2007							
			Gidycz et al., 1991							
			Gidycz et al., 1993							
			Gidycz et al., 2008							
			Gilboa-Schechtman et al.,2001							
			Goldmann et al., 2011							
			Gutner et al., 2006							
			Hagen, 1998							
			Halligan et al., 2003							
			Hapke et al., 2006							
			Harris et al., 2002							
			Heidt et al., 2005							
			Hidaka et al., 2014							
			Hossain et al., 2010							
			Johnson et al., 2010							
			Kalichman et al., 2001							
			Kaltman et al., 2005							
			Kaminer et al., 2008							
			Kaysen et al., 2011							
			Keller et al., 2006							
			Kelley, 2009							
			Kelley et al., 2009							
			Kendall-Tackett et al., 2013							
			Kennedy et al., 2014							
			Kessler et al., 1999							
			Kievit, 1999							
			Kilpatrick et al., 1992							
			Kilpatrick et al., 1981							
			Kilpatrick et al., 2007							
			Kimerling et al., 2007							
			King, 2003							
			Kunst et al., 2011							
			Lancaster et al., 2014							
			Lang et al., 2003							
			Larimer et al., 1999							
			Laura, 2012							
			Laws et al., 1996							

APPENDIX A. Characteristics of included review studies (included)

	Synthesis	Timeframe	Included studies	Region	Design	Sample	ASA context	Changes	R/P factor	Quality
			Layman et al., 1996<br>Lexington et al., 2006<br>Lindquist et al., 2013<br>Littleton et al., 2012<br>Littleton et al., 2014<br>Logie et al., 2014<br>Loshek, 2014<br>Lown et al., 2001<br>Lueger-Schuster et al., 2012<br>Martin et al., 2008<br>Martinso, 2013<br>Masho et al., 2007<br>Matich-Maroney, 1996<br>***McAuslan, 1998***<br>***McCutcheon et al., 2010***<br>Messman-Moore et al., 2005<br>Messman-Moore et al., 2008<br>Messman-Moore et al., 2000<br>Meyerson, 2002<br>Miller et al., 1995<br>***Moss et al., 1990***<br>Nayak et al., 2012<br>Norris, 1992<br>Nosek et al., 2006<br>Orchowski, 2009<br>Orth et al., 2004<br>Orth et al., 2008							
**Dworkin et al. (2019)**	Narr, Num	Ns	***Ahrens et al., 2009***<br>Ammerman et al., 2013<br>***Borja et al., 2006***<br>Dardis, 2011<br>DeCou et al., 2016<br>***Deitz et al., 2015***<br>Dunmore et al., 2001<br>Elklit et al., 2013<br>***Jacques-Tiura et al., 2010***<br>Kelley, 2013<br>Kelley et al., 2013<br>Littleton, 2003<br>***Littleton, 2010***<br>Lopez, 2016<br>***McAuslan, 1998***<br>Menatti, 2016<br>Routbort, 1998<br>Staples et al., 2016	United States, Denmark	Quanti	4045 women and men, age ns, clinical, community and college samples	Any ASA	I	MM, E	6

APPENDIX A. Characteristics of included review studies (included)

	Synthesis	Timeframe	Included studies	Region	Design	Sample	ASA context	Changes	R/P factor	Quality
			Ullman, 2000, 2004<br>Varkovitzky, 2008							
**Dworkin et al. (2021)**	Narr, Tab, Num, Graph	Until 2020	Armour et al., 2012<br>Darves-Bornoz et al., 1998<br>Foa et al., 1995<br>Frazier, 1988a, 1988b<br>***Frazier et al., 2001***<br>Gilboa-Schectman et al., 2001<br>Gilmore et al., 2019a, 2019b<br>***Gutner et al., 2006***<br>Hansen et al., 2017<br>Hyland et al., 2016<br>***Kaysen et al., 2010***<br>***Kaysen et al., 2011***<br>Khadr et al., 2018<br>***Koss et al., 2004***<br>Machado et al., 2011<br>Mathews et al., 2013<br>Miller et al., 2005<br>Nickerson et al., 2013<br>Steenkamp et al., 2012<br>Quidé et al., 2018<br>***Resick, 1988***<br>Resnick et al., 2007a, 2007b<br>Rothbaum et al., 1992<br>Rothbaum et al., 2012<br>Ulirsch et al., 2014<br>Walsch et al., 2017	United States, Denmark, France, England, Brasil, South-Africa	Quanti	2106 women and men, age ns, community and clinical samples	Any ASA	I	I, C	7
**Elderton et al. (2017)**	Narr, Tab, Graph	Ns	***Borja et al., 2006***<br>Cole et al., 2010<br>***Frazier et al., 2001, 2004***<br>***Kennedy et al., 1998***<br>***Thompson, 2000***	United States, United Kingdom	Quanti and quali	466 women, age ns, clinical, community and student samples	Sexual interpersonal violence	+	C, I, A, MM, E	6
**Feldner et al. (2007)**	Narr, Tab	Ns	Hapke et al., 2005<br>Lemon et al., 2002<br>Weaver et al., 2003	Germany, United States,	Quanti	5698 women and men, 18-64 years, community and clinical samples	Any ASA	I	A	5
**Finneran et al. (2012)**	Narr, Tab	1990-2011	Pantalone, 2011<br>Wong, 2010<br>Brastein, 2006<br>Feldman, 2007<br>Greenwood, 2002<br>***Kalichman, 2001***<br>Kalichman, 1995	United States	Quanti	>5278 MSM, 18-59 years, clinical and community samples	SIPV	I		5

APPENDIX A. Characteristics of included review studies (included)

	Synthesis	Timeframe	Included studies	Region	Design	Sample	ASA context	Changes	R/P factor	Quality
**Forkus et al. (2021)**	Narr, Tab	Until 2018	Backhaus et al., 2016<br>Bartlett et al., 2018<br>Beckman et al., 2018<br>Blais et al., 2017<br>Booth et al., 2011, 2012<br>Breland et al., 2018<br>Brown et al., 2015<br>Bryan et al., 2015<br>Butterfield et al., 1998<br>Cichowski et al., 2017<br>Creech et al., 2014<br>***DiMauro et al., 2018***<br>Fillo et al., 2018<br>***Forman-Hoffman et al., 2012***<br>***Frayne et al., 2003***<br>Gilmore et al., 2016<br>Gobin et al., 2015<br>Godfrey et al., 2015<br>***Gradus et al., 2008***<br>Gradus et al., 2013a, 2013b<br>Griffith, 2017<br>Hahn et al., 2015<br>***Hankin et al., 1999***<br>Harned et al., 2002<br>Hoggatt et al., 2015<br>Jenkins et al., 2015<br>Katz et al., 2012<br>Kimerling, 2010<br>Kimerling et al., 2016<br>Klingensmith et al., 2014<br>***Maguen et al., 2012***<br>Monteith et al.., 2015, 2016a, 2016b<br>Pavao et al., 2013<br>Rosellini et al., 2017<br>Rowe et al., 2009<br>Ryan et al., 2015<br>Schry et al., 2015<br>Schuyler et al., 2017<br>Stahlman et al., 2014, 2015<br>Strauss et al., 2011<br>Tiet et al., 2015<br>***Turchik et al., 2012***<br>Yalch et al., 2018	United States	Quanti	8876302 female and male veterans, mean age 25.7-55 years	Military sexual violence	I	I	7

APPENDIX A. Characteristics of included review studies (included)

	Synthesis	Timeframe	Included studies	Region	Design	Sample	ASA context	Changes	R/P factor	Quality
**Galatzer-Levy et al. (2018)**	Narr, Tab, Num	Before 2016	Armour et al., 2011	Denmark, United States	Quanti	374 respondents, no further info stated	Non-military sexual violence		C	4
**Gallegos et al. (2021)**	Narr, Tab	1946-2017	***Kelly et al., 2011***	Ns	Quanti	135 female veterans, age ns	Military sexual violence	I		7
**Gielen et al. (2007)**	Narr, Tab	1997-2006	***Collins et al., 2005***	United States	Quanti	2901 respondents from community sample, no further info stated	SIPV	I	I, MM	6
**Godier-McBard & Jones (2020)**	Narr, Tab	Until 2019	DuBois et al., 1998<br>Faley et al., 2006<br>Goyer et al., 1984<br>Leskela et al., 2001<br>Magley et al., 1999<br>Monteith et al., 2019<br>O'Brien et al., 2015<br>Stockdale et al., 1999	United States	Quanti en quali	37394 female and male veterans, age 18-31	Military same-sex sexual violence	I, IP	I, A	6
**Gong et al. (2019)**	Narr, Tab	Until 2018	Blayney et al., 2016<br>Brown et al., 2009a, 2009b<br>Jaffe et al., 2017<br>***Kaysen et al., 2006***<br>***Kaysen et al., 2010***<br>***Kaysen et al., 2011***<br>***Littleton et al., 2009***<br>Masters et al., 2015<br>***Peter-Hagene et al., 2015***<br>Peter-Hagene et al., 2018<br>Zinzow et al., 2010a, 2010b, 2012	United States	Quanti	8885 women and men, 19-76 years, community, clinical and college samples	Any ASA		I, C, A	7
**Goodman et al. (1997)**	Narr, Tab	Ns	Bryer et al., 1987<br>Jacobson et al.,1987<br>Cole, 1988<br>Muenzenmaier et al., 1993<br>Goodman et al., 1995<br>Cloitre et al., 1996	Ns	Quanti	956 women, age ns, clinical samples	Any ASA	I		4
**Guggisberg et al. (2021)**	Narr, Tab, Graph	2010-2020	Anderson et al., 2018<br>Catabay et al., 2018<br>Foster et al., 2014<br>Kelley et al., 2015	United States, Europe	Quanti, quali, mixed	354 female and male survivors, age ns	Any ASA	+		7

APPENDIX A. Characteristics of included review studies (included)

	Synthesis	Timeframe	Included studies	Region	Design	Sample	ASA context	Changes	R/P factor	Quality
**Halstead et al. (2017)**	Narr, Tab	2010-2015	***Littleton, 2010***<br>Smith et al., 2013<br>***Miller et al., 2011***<br>***Zinzow et al., 2011***<br>Koo et al., 2015<br>Orchowski et al., 2012<br>***Orchowski et al., 2013***	United States	Quanti	1438 female survivors, female and male disclosure recipients, 18-50 years, student samples	Any ASA	IP	MM	5
**Heerde et al. (2016)**	Narr, Tab	1990-2013	Tyler et al., 2013<br>Whitbeck et al., 2004	Ns	Quanti	677 homeless women and men, 14-21 years	Any ASA	I		6
**Hellman (2014)**	Narr, Tab	Since 2003	***Ben-Ezra et al., 2010***<br>***Patterson et al., 2009***<br>***Ullman et al., 2009***<br>***Ullman et al., 2007***<br>***Ahrens et al., 2010***<br>Bilette et al., 2008<br>***Borja et al., 2006***<br>Bryant-Davis et al., 2011<br>Campbell et al., 2010<br>Fetchenhauer et al., 2005<br>***Frazier, 2003***<br>***Frazier et al., 2005***<br>***Frazier et al., 2004***<br>***Koss et al., 2004***<br>Littleton et al., 2011<br>Littleton, 2007<br>***Miller et al., 2010***<br>***Ullman et al., 2011***	Ns	Quanti and quali	4267 women, *M* = 32 years, clinical, community and student samples	Any ASA		C, I, MM, (Ma)	5
**Kahsay et al. (2020)**	Narr, Tab	Until 2018	Othman et al., 2018<br>Fatema, 2017<br>Hussein et al., 2015<br>Ali et al., 2015<br>***Mustaq et al., 2015***<br>Khan et al., 2015<br>Suhaila et al., 2012<br>Shiao et al., 2010<br>***Celik et al., 2007***<br>Gunnarsdottir et al., 2006<br>Kisa et al., 2002<br>Matsuoka et al., 2001<br>Kisa et al., 1996<br>Dan et al., 1995<br>Libbus et al., 1994	United States, Japan, Turkey, Iceland, Malaysia, Taiwan, Pakistan, Egypt, Kenya, Bangladesh	Quanti	4479 female nurses, age ns	Sexual harrassment at the workplace	I, IP		7

APPENDIX A. Characteristics of included review studies (included)

	Synthesis	Timeframe	Included studies	Region	Design	Sample	ASA context	Changes	R/P factor	Quality
**Kennedy et al. (2018)**	Narr	2000-2015	***Frazier et al., 2005***<br>***Orchowski et al., 2013a***<br>Walsh et al., 2011<br>Breitenbecher, 2006<br>Koss et al., 2002<br>Hassija et al., 2013<br>***Miller et al., 2010***<br>Perilloux et al., 2014<br>***Koss et al., 2004***<br>***Frazier, 2003***<br>Katz et al., 2010<br>Miller et al., 2007<br>Heath et al., 2011<br>Wang, 2011<br>Carretta et al., 2015<br>***Patterson et al., 2009***<br>Sturza et al., 2005<br>Jones et al., 2009<br>Weiss, 2010<br>Hamby, 2008<br>***Zinzow et al., 2011***<br>***Miller et al., 2011***<br>***Campbell, 2008***<br>Kennedy et al., 2012<br>Ahrens et al., 2012<br>***Ahrens et al., 2009***<br>Ahrens et al., 2007<br>Filipas et al., 2001<br>***Ahrens et al., 2010***<br>***Sigurvinsdottir et al.,2015***<br>Starzynski et al., 2014<br>Ullman, 2007<br>Ullman et al., 2001<br>***Ullman et al., 2014***<br>Mason et al., 2009<br>Orchowski et al., 2015<br>Orchowski et al., 2013b<br>Campbell, Wasco et al., 2001<br>Campbell, Ahrens et al.,2001<br>Campbell, 2005<br>***Campbell et al., 2006***<br>Campbell et al., 2005<br>***Jacques-Tiura et al., 2010***<br>***Borja et al., 2006***<br>Ahrens, 2006<br>Littleton et al., 2008<br>***Deitz et al., 2015***	Ns	Quanti, quali and mixed	Samples of women, no further info stated	Any ASA		C, I,<br>MM,<br>E,<br>(Ma)	5

APPENDIX A. Characteristics of included review studies (included)

	Synthesis	Timeframe	Included studies	Region	Design	Sample	ASA context	Changes	R/P factor	Quality
			Littleton, 2010<br>Littleton et al., 2006<br>***Littleton et al., 2009***<br>**Ullman, Filipas, Townsend et al., 2006a**<br>Ullman et al., 2008<br>Relya et al., 2014<br>Long et al., 1997<br>***Ullman, Townsend, Filipas et al., 2007***<br>***Peter-Hagene et al., 2015***<br>***Ullman et al., 2011***<br>***Ullman et al., 2009***<br>***Najdowski et al., 2009***<br>Starzynski et al., 2005<br>***Ullman, 2014***<br>Ullman, Townsend, Filipas et al., 2000b<br>***Ullman et al., 2014***<br>Ullman et al., 2008<br>***Gibson et al., 2001***<br>***Filipas et al., 2006***<br>Neville et al., 2004							
**Klein & Martin (2021)**	Narr, Tab	From 2000 unwards	Buchanan et al., 2009<br>Cantor et al., 2015<br>Huerta et al., 2006<br>Jirek et al., 2018<br>Kalof et al., 2001<br>McGinley et al., 2016<br>Rospenda et al., 2000<br>Shinsako et al., 2001<br>Wolff et al., 2017<br>Yoon et al., 2010	United States	Quanti	164337 women and men, 17-52 years, student samples	Sexual harrassment in college	I	I	5
**Klein et al. (2022)**	Narr	Since 2000	Backhaus et al., 2019<br>Blosnich et al., 2012<br>***Blosnich et al., 2011***<br>Holmes et al., 2021<br>Kammer-Kerwick et al., 2019<br>McCauley et al., 2020<br>Moschella et al., 2020<br>***Parr, 2020***<br>Potter et al., 2020	United States	Quanti, quali, mixed	234733 LGBTQ+ minority college students, age ns	SIPV	I,+	I	6

APPENDIX A. Characteristics of included review studies (included)

	Synthesis	Timeframe	Included studies	Region	Design	Sample	ASA context	Changes	R/P factor	Quality
**Knight et al. (2022)**	Narr, Tab	2000-2019	Ainamo, 2017<br>Cecchet et al., 2014<br>Chilaka, 2019<br>Ginesini, 2018<br>Herdiana et al., 2019<br>Hicle et al., 2017<br>Kometiani et al., 2020<br>O'Brien, 2018	United States, Italy, Kenia, Indonesia	Quanti en quali	Sample of women and Men survivors of sexual human trafficking, no further details specified	Human trafficking		I, MM, E	7
**Kouvelis & Kangas (2021)**	Narr, Tab	Until 2020	Keshet et al., 2019<br>Clifford et al., 2020a, 2020b	Israël, United Kingdom	Quanti	833 women, 18-55 years, clinical and community samples	Any ASA	I	I	7
**Kouyoumdjian et al. (2013)**	Narr, Tab	Until 2011	Reading et al., 2009	Canada	Quali	Sample of HIV positive aboriginal women, no further details specified	SIPV	I		6
**Langdon et al. (2017)**	Narr, Tab	Until 2015	Bedard-Gilligan et al., 2011<br>Corbin et al., 2001<br>***Gradus et al., 2008***<br>Han et al., 2013<br>***Hankin et al., 1999***<br>***Kaysen et al., 2006***<br>***Kimerling et al., 2010***<br>Kilpatrick et al., 1997<br>Larimer et al., 2009<br>***Littleton et al., 2009***<br>***Maguen et al., 2012***<br>Marx et al., 2000<br>McCauley et al., 2009<br>***McCauley et al., 2010***<br>Messman-Moore et al., 2009<br>Miranda et al., 2002<br>***Najdowski et al., 2009***<br>***Nguyen et al., 2010***<br>***Peter-Hagene et al., 2015***<br>Resnick et al., 2013<br>Stappenback et al., 2015<br>Timko et al., 2008<br>Ulibarri et al., 2015<br>***Ullman et al., 2009***<br>Walsh et al., 2014<br>Zinzow et al., 2012	Ns	Quanti	381531 women and men, mean age 19.2-35.9, clinical, community, college and military samples	Any ASA	I	I	5
**Larijani et al. (2015)**	Narr, Tab	2000-2011	Walker et al., 1996	United States	Mixed	462 women, 18-45 years, clinical sample	Any ASA	I		6

APPENDIX A. Characteristics of included review studies (included)

	Synthesis	Timeframe	Included studies	Region	Design	Sample	ASA context	Changes	R/P factor	Quality
**Lim et al. (2022)**	Narr	Until 2019	***Buchanan et al., 2009***<br>Buchanan et al., 2018<br>Crisanti et al., 2011<br>Devries et al., 2011<br>Dir et al., 2018<br>Hahm et al., 2012, 2017<br>Jewkes, Jama- Shai, et al., 2017<br>Kelly-Hanku et al., 2015<br>McLaughlin et al., 2019<br>Nemoto et al., 2004<br>***Nguyen et al., 2010***<br>Nusbaum et al., 2005<br>Pape et al., 2016<br>Tummala-Narra et al., 2019<br>Yoshihama, 2001a	United States, Papua New Guinea	Quanti, quali, mixed	Asian American, Native Hawaiian, and Pacific Islander adults, no further details specified	Non-SIPV	I, IP		5
**LoGiudice (2017)**	Narr, Tab	1990-2016	Scherdtfeger et al., 2009	Ns	Quali	10 women, 21-38 years, clinical sample	Any ASA		C	5
**Lombardi et al. (2023)**	Narr, Tab, Num, Graph	Until 2020	Cohen et al., 2002<br>***Littleton et al., 2015***<br>Meltzer-Brody et al., 2013<br>Sorbo et al., 2014	Canada, United States, The Netherlands, Norway	Quanti	54351 women, no further details specified	Any ASA	I		6
**Madowitz et al. (2015)**	Narr, Tab	2004-2014	***Faravelli et al., 2004***<br>Hepp et al., 2007<br>Fischer et al., 2010<br>Reyes-Rodriquez et al., 2011<br>***Dubosc et al., 2012***	Ns	Quanti	1895<br>Women, 17-50 years, community	Any ASA	I		6
**Maman et al. (2000)**	Narr, Tab	Ns	Choi et al., 1998<br>***Kalichman et al., 1998***<br>Wingood et al.,1998	United States	Quanti	2320 women and men, 18-49 years, community samples	SIPV	I		4
**Mauritz et al. (2013)**	Narr, Tab, Num, Graph	1980-2010	Goldberg et al., 2005<br>Carlier et al., 2000<br>Oquendo et al., 2005<br>Beattle et al., 2009<br>***Calhoun et al., 2007***<br>Kilcommons et al., 2005<br>Lommen et al., 2009<br>Resnick et al., 2003<br>Yen et al., 2002<br>Cusack et al., 2006<br>Goodman et al., 1999<br>Lu et al., 2008<br>***Mueser et al., 1998***<br>Mueser et al., 2004	United States, Brasil, The Netherlands, Ireand	Quanti	2942 women and men, M = 33-46 years, clinical samples	Any ASA	I		7

APPENDIX A. Characteristics of included review studies (included)

	Synthesis	Timeframe	Included studies	Region	Design	Sample	ASA context	Changes	R/P factor	Quality
**Mazza et al. (2021)**	Narr	2011-2021	***Paulson et al., 2020***	Ns	Quanti	Sample of women, no details specified	SIPV	I		5
**Messman-Moore et al. (2003)**	Narr, Tab	Ns	***Cloitre et al., 1997***<br>Wyatt et al., 1995<br>***Gidycz et al., 1995***<br>Himelein, 1995	United States	Quanti	1308 respondents, gender ns, 18-36 years, community samples	Any ASA	I, IP	C	4
**Meyer, et al. (2011)**	Narr, Tab	1990 - 2010	***Collins et al., 2005***<br>Stockman et al., 2010<br>Hamburger et al., 2004<br>Stoner et al., 2008	Ns	Quanti	9508 women and men, age ns, community samples	Any ASA	I		6
**Molstad et al. (2023)**	Narr, Tab	Until 2021	Baker et al., 2016<br>***Jordan et al., 2014***<br>Mengo et al., 2016<br>***Griffin et al., 2012***<br>Wood et al., 2020<br>Kaufman et al., 2019<br>Brewer et al., 2018<br>Brewer et al., 2019<br>Baynard et al., 2020<br>Stermac et al., 2020<br>Bonomi et al., 2018<br>Nwadigwe, 2007<br>Tremblay et al., 2008	United States, Nigeria	Quanti, quali, mixed	191540 women and men, age ns, student samples	Any ASA	I	C	7
**Nicholas et al. (2022)**	Narr, Tab	2010-2022	***Parr, 2020***	United States	Quanti	14908 men, mean age = 21.5, student sample	Any ASA	I	I	7
**Normann et al. (2020)**	Narr, Tab, Graph	Until 2019	Boyce et al., 2017	India	Quanti	10469 women, 20-29 years, Community sample	SIPV	IP		6
**Ozer et al. (2003)**	Narr, Tab	1980-2000	Kramer et al., 1991<br>Resnick et al., 1995	Ns	Quanti	Clinical samples of women, no further details specified	Any ASA		MM, C	7
**Paras et al. (2009)**	Narr, Tab, Num, Graph	1980-2008	Rapkin et al., 1990<br>Reinhard, 2004<br>***Salmon et al., 2003***<br>Walker et al., 1995<br>Walker et al., 1997<br>Romans et al., 2002	United Kingdom, New Zealand	Quanti	1253 women and Men, mean age 29-47 years, clinical and community samples	Any ASA	I		7
**Paulson (2020)**	Narr, Tab	Unril 2021	Budhathoki, 2013<br>Dennis, 2013<br>Tho Tran, 2018<br>Pellowski, 2019	South-Asia, Canada, Vietnam, South-Africa, Hong	Quanti	10853 pregnant women or women with children, mean age 16-49 years, clinical and community samples	SIPV	I		5

APPENDIX A. Characteristics of included review studies (included)

	Synthesis	Timeframe	Included studies	Region	Design	Sample	ASA context	Changes	R/P factor	Quality
			Tiwari, 2008<br>Zhang, 2012<br>Ludermir, 2010<br>Rogathi, 2017<br>Tho Nhi, 2019<br>Fisher, 2013<br>Okafor, 2018	Kong, China, Brazil, Tanzania						
**Pebole et al. (2021)**	Narr, Tab	1980-2020	Hesdon, 2003<br>***Frayne, 2003***<br>Hollander, 2010<br>Concepcion, 2005	United States	Quanti en quali	4315 women and men, 18-54 years, student, community and military samples	Any ASA	I	I	5
**Peterson et al. (2011)**	Narr, Tab	1984-2009	***Elliott et al., 2004***<br>Krahe et al., 2003<br>Mezey et al., 1989<br>Walker et al., 2005a<br>Walker, Archer et al., 2005b<br>Banyard et al., 2007<br>Lydum et al., 1999<br>Struckman-Johnson, 1988<br>Struckman-Johnson et al., 1994<br>O'sullivan et al., 1998<br>***Heidt et al., 2005***<br>***Kalichman et al., 2001***<br>***Kalichman et al., 1995***<br>Kalichman et al., 2002<br>Ratner et al., 2003<br>Kang et al., 2005<br>Libscomb et al., 1992<br>Struckman-Johnson et al., 2006<br>Struckman-Johnson et al., 1996<br>Wolff et al., 2009<br>Kimerling et al., 2002<br>***Coxell et al., 1999***<br>King et al., 2002<br>Stermac et al., 1996	United States, Canada, Germany, United Kingdom	Quanti	32293 women and men, age ns, clinical, community, student, military and prison samples		I, IP	I, A, E, (Ma)	5
**Pulverman & Creech (2021)**	Narr, Tab	Until 2018	***Turchik et al., 2012***<br>***O'Brien et al., 2008***<br>***Garneau-Fournier et al., 2018***<br>***Campbell et al., 2006***<br>***Blais et al., 2018***<br>McCall et al., 2000	United States	Quanti	58611female veterans, age ns	Any ASA	I	I	6
**Pulverman et al. (2019)**	Narr, Tab	Until 2018	***DiMauro et al., 2018***<br>***Garneau-Fournier et al., 2018***	Ns	Quanti	61016 female veterans, age ns	Any ASA	I	I, C	6

APPENDIX A. Characteristics of included review studies (included)

	Synthesis	Timeframe	Included studies	Region	Design	Sample	ASA context	Changes	R/P factor	Quality
			McCall-Hosenfeld et al.,2009<br>***O'Brien et al., 2008***<br>Skinner et al., 2000<br>***Turchik et al., 2012***							
**Rani et al. (2022)**	Narr	Until 2020	Bohra et al., 2015<br>***Goodman et al., 1997***<br>Harris, 1994<br>Hegarty, 2011<br>Hodges et al., 2004<br>Howard et al., 2010<br>Indupalli et al., 2014<br>***Khalifeh et al., 2015***<br>Xie, 2013	Ns	Ns	Female survivors with severe mental illness	SIPV	I, IP	I, C	4
**Ribeiro et al. (2009)**	Narr, Tab	Until 2009	Ilianes et al., 2007<br>Ludemir et al., 2008<br>Patel et al, 2005<br>***Naved et al., 2008***	Chile, Brazil, India, Bangladesh	Quanti	Community samples of women 15-19 years, sample size ns	Any ASA	I		5
**Romans et al. (2008)**	Narr, Tab, Graph	1966-2005	Talley et al., 1998<br>Reilley et al., 1999<br>Hobbis et al., 2002<br>***Salmon et al., 2003***<br>Williams et al., 2004<br>Anderberg et al., 2000	Australia, United Kingdom	Quanti	2147 women and men, age ns, clinical and community	Any ASA	I		7
**Salim et al. (2022)**	Narr, Tab, Graph	Until 2021	***Blosnich et al., 2011***<br>Drabble et al., 2013<br>Hequembourg et al., 2013<br>Hughes et al., 2010<br>Kelley et al., 2010a, 2010b<br>Long et al., 2007<br>McConnell et al., 2019<br>Rhew et al., 2017<br>Salim et al., 2021<br>***Sigurvinsdottir et al., 2015***, 2016a, 2016b	United States	Quanti	4205 women, 18-70 years, community, clinical and college samples	Any ASA	I	I, MM, C	6
**Scoglio et al. (2021)**	Narr, Tab, Graph	1985-2017	Miron et al., 2014	Ns	Quanti	541female college students	Any ASA	I		6
**Seth et al. (2013)**	Narr, Tab	2000-2013	Alleyne et al., 2011<br>***Collins et al., 2005***<br>***Lang et al., 2011***<br>Silverman et al., 2001	United States	Quanti	8555 women and men, 14-24 years, community and student samples	SIPV	I	I, MM	5

APPENDIX A. Characteristics of included review studies (included)

	Synthesis	Timeframe	Included studies	Region	Design	Sample	ASA context	Changes	R/P factor	Quality
**Shamblaw et al. (2019)**	Narr, Tab, Num	1980-2016	Hedin et al., 1999<br>Lewis et al., 2017<br>Lovisi et al., 2005<br>Mahenge et al., 2013<br>Martin et al., 2006<br>Shneyderman et al., 2013	Ns	Quanti	2857 women, no further details specified	Any ASA	I		6
**Sinko et al. (2022)**	Narr, Tan, Graph	Until 2020	dos Reis et al., 2016<br>***Duma et al., 2007***<br>Farrell, 1996<br>Ranjbar et al., 2013<br>Sinko et al., 2019<br>Smith et al., 2001<br>***Thompson, 2000***<br>Zraly et al., 2010	United States, Rwanda, United Kingdom, Brazil, South-Africa	Quali	Female survivors, Mean age 31.59-39.75, no further details specified	Any ASA		I, MM, E	7
**Sparrow et al. (2017)**	Narr, tab	Ns	Mercado, 2015<br>Dichter, 2014	United States, United Kingdom	Quanti	618 women, age ns, clinical and military samples	SIPV	I	A	7
**Spencer et al. (2023)**	Narr, Num	2000-2019	Ns	United States	Quanti	333722 college students, no further details specified	College sexual assault	I, IP		5
**Steine et al. (2012)**	Narr, Tab	Until 2010	***Frank et al., 1984***<br>Loncar et al., 2010	Until 2010	Quanti	150 women and men, 14-47 years, clinical and military samples	Any ASA	I		5
**Stewart et al. (2019)**	Narr, Tab, Graph	Until 2016	Amstadter et al., 2009<br>Bryan et al., 2016<br>***Burgess et al., 1979***<br>Collibee et al., 2014<br>***Duma et al., 2007a, 2007b***<br>***Ellis et al., 1981***<br>Gidycz et al., 2007<br>***Griffin et al., 2012***<br>***Jordan et al., 2014***<br>***Lang et al., 2011***<br>Lindgren et al., 2012<br>***McCauley et al., 2010***<br>McMullin et al., 2006<br>Messman-Moore et al., 2015<br>Mouliso et al., 2012<br>***Najdowski et al., 2009***<br>Nowotny et al., 2013<br>Parks et al., 2014<br>Resick et al., 1981<br>Testa et al., 2000<br>***Testa et al., 2007***, 2010	United States, South Africa	Quanti	Samples of female survivors, mean age 31.59-39.75	Any ASA	I, IP		6

APPENDIX A. Characteristics of included review studies (included)

	Synthesis	Timeframe	Included studies	Region	Design	Sample	ASA context	Changes	R/P factor	Quality
			Tsai et al., 2016<br>Ullman, 2016<br>Ullman et al., 2005							
**Stockman et al. (2013)**	Narr, Tab	Since 2000	Dunkle, 2004<br>El Bassel, 2007<br>Hoffman, 2006<br>Morokoff, 2009<br>Panchanadeswaran, 2010<br>Simoni, 2004	Ns	Quanti	3087 women and men, 16-67 years, clinical, community and student samples	Any ASA	I		6
**Tolin et al. (2008)**	Narr, Tab	1980-2007	Ns	Ns	Quanti	Samples of women and men, no further details specified	Any ASA		I	5
**Trevillion et al. (2012)**	Narr, Tab, Num, Graph	2007-2011	Deyessa, 2009<br>Weizmann-Henelius, 2004<br>Najavits, 2004	Ethiopia, Finland, United States	Quanti	2144 women, age ns, clinical and community samples	SIPV	I		7
**Ullman (1999)**	Narr, Tab	Ns	***Atkeson et al., 1982***<br>Burgess et al., 1978<br>***Cohen et al., 1987***<br>Davis et al., 1991<br>Kimerling et al., 1994<br>Kaniasty et al., 1992<br>***Moss et al., 1990***<br>Popiel et al., 1985<br>***Resick, 1988***<br>Ruch et al., 1983<br>Sales et al., 1984<br>Ullman, 1996a, b, c, d<br>West et al., 1987<br>Wyatt et al., 1990	Ns	Quanti	1764 women, age ns, clinical and community samples	Any ASA		I, MM, E	4
**Ullman (2004)**	Narr	Until 2002	***Burnam et al., 1988***<br>Kilpatrick et al., 1985<br>***Davidson et al., 1996***<br>Ullman et al., 2002<br>Nelson et al., 1994<br>Coker et al., 2000<br>Manetta, 1999<br>Stepakoff, 1998<br>Thakkar et al., 2000<br>Peters et al., 1995<br>Yoder, 1999	United States	Quanti	>7398 women, age ns, clinical and community samples	Any ASA	I	C, I, A, MM	6

APPENDIX A. Characteristics of included review studies (included)

	Synthesis	Timeframe	Included studies	Region	Design	Sample	ASA context	Changes	R/P factor	Quality
**Ulloa et al. (2016)**	Narr, Tab	Since 1980	***Borja et al., 2006***<br>Burt et al., 1987<br>Cole, 2008<br>***Frazier et al., 1994***<br>***Frazier et al., 2001, 2004***<br>Frazier et al., 2006<br>Grubaugh et al., 2007<br>Guerette et al., 2007<br>Gwynn, 2008<br>***Kennedy et al., 1998***<br>Kleim et al., 2009<br>Kunst, 2010<br>Shakespeare et al., 2009<br>***Thompson, 2000***<br>***Ullman, 2014***	Ns	Quanti, quali and mixed	3463 women and men, 18-60 years, no further details specified	Any ASA	I, +	C, I, A, MM, E	5
**van Berlo et al. (2000)**	Narr	Since 1993	Feldman-Summers et al., 1979<br>Orlando et al., 1983<br>Norris et al., 1981<br>Koss et al., 1988<br>Kilpatrick et al., 1988<br>Becker et al., 1982<br>Nadelson et al., 1982<br>Bartoi et al., 1998<br>Letourneau et al., 1996<br>Burgess et al., 1979<br>Ellis et al., 1980<br>Dahl, 1993<br>Van Berlo, 1999	United States, Norway	Quanti	1865 women, age ns, clinical, community and student samples	Any ASA	I	C, I, A, MM	5
**Vitek & Yeater (2021)**	Narr, Tab	Until 2019	***Gidycz et al., 2008***<br>Katz et al., 2008<br>Thelen et al., 1998<br>Georgia et al., 2018<br>Messman-Moore et al., 2000<br>Miner et al., 2006	Ns	Quanti	2195 women and heterosexual couples, age ns, college and community samples	Any ASA	IP		
**Wadsworth, et al. (2013)**	Narr, Tab	2001-2012	***Kimerling et al., 2010***<br>Miner, 2006<br>Molina, 2007<br>Ramos, 2004<br>Street, 2008<br>Teitelman, 2011<br>Temple, 2007<br>Kaukinen, 2005<br>Plichta, 2001	Ns	Quanti and quali	188105 women and men, age ns, community and military samples	Any ASA	I	C, I, A	7

APPENDIX A. Characteristics of included review studies (included)

	Synthesis	Timeframe	Included studies	Region	Design	Sample	ASA context	Changes	R/P factor	Quality
**Walker et al. (2017)**	Num, Graph	Ns	***Balsam et al., 2011***<br>Banyard et al., 2011<br>Barett-Model et al., 2010<br>Brenner et al., 2015<br>***Campbell et al., 2008***<br>***Cloitre et al., 2002***<br>Davis et al., 2004<br>de Haas et al., 2012<br>Dietrich, 2003<br>Fergusson et al., 1997<br>Fortier et al., 2009<br>Heidt et al., 2009<br>Hughes et al., 2010<br>Janowski et al., 2002<br>***Kessler et al., 1999***<br>Lehn, 2003<br>Leonard, 1991<br>***Maker et al., 2001***<br>Meija et al., 2015<br>Messman-Moore, 1991<br>Messman-Moore et al., 2007<br>Messman-Moore et al., 2002<br>Morris et al., 2003<br>***Murphy et al., 1988***<br>Pantalone et al., 2014<br>***Paul et al., 2001***<br>***Proulx et al., 1995***<br>Randall et al., 1995<br>Reese-Weber et al., 2001<br>Reig, 1998<br>Rubien, 1995<br>Schroeder, 2005<br>Simoni et al., 2002<br>Stevenson et al., 1992<br>Walsch, 2009<br>Walsch et al., 2013<br>Weinberg, 2008<br>Whetsell, 1990<br>***Wyatt et al., 1992***<br>Yiaslas et al., 2014<br>Zinzow et al., 2008	Ns	Quanti	No info stated	Adolescent sexual violence	I		7
**Wilness et al. (2007)**	Narr, Num	Ns	Ns	Ns	Quanti	>45880 respndents, no further info stated	Sexual harrassment at the workplace	I		6

APPENDIX A. Characteristics of included review studies (included)

	Synthesis	Timeframe	Included studies	Region	Design	Sample	ASA context	Changes	R/P factor	Quality
**Wright et al. (2022)**	Narr, Tab	2003:2020	Gilroy et al., 2015<br>Holt et al., 2003<br>Howard et al., 2003<br>McFarlane et al., 2005<br>***Ullman et al., 2006***	United States	Quanti	2396 women, 18-52 years, community, clinical and college samples	SIPV		MM, E	7
**Zarchev et al. (2021)**	Nar, Num, Graph	Until 2020	Afful, 2010<br>Amir, 2012<br>Anderson, 2016<br>Armstrong, 2014<br>Barry, 2011<br>Bengtsson-tops, 2012<br>Bone, 2018<br>Bonin, 2000<br>Braitstein, 2003<br>Branstetter, 2008<br>***Calhoun, 2007***<br>Chang, 2011<br>Clancy, 2006<br>Coverdale, 2000<br>Daigre, 2015<br>Dammeyr, 2018<br>Dansky, 1996<br>De Oliveira, 2012<br>De Waal, 2017<br>***Dworkin, 2017***<br>Fiorentine, 1999<br>Goodman, 2001<br>Guimaraes, 2017<br>Heru, 2006<br>Hutchings, 1993<br>Jakubczyk, 2014<br>Kalokhe, 2012<br>Kamperman, 2014<br>***Khalifeh, 2015***<br>Lapp, 2005<br>Lipschitz, 1996<br>Llario, 2019<br>Mckeganey, 2017<br>Moncrieff, 1996<br>***Mueser, 1998***<br>Pan, 2007<br>Pearce, 2008<br>Platt, 2017<br>Ruiz-Perez, 2018<br>Shack, 2004	High-income Western countries, India, Taiwan, Brazil	Quanti	45172 men with a mental illness diagnosis, age ns	Any ASA	I		6

APPENDIX A. Characteristics of included review studies (included)

	Synthesis	Timeframe	Included studies	Region	Design	Sample	ASA context	Changes	R/P factor	Quality
			Teplin, 2005<br>Tham, 1995<br>Tiet, 2006<br>Villano, 2007<br>Yen, 2002							

Note. Narr = Narrative synthesis, Tab = Tabular synthesis, Num = Numerical synthesis; Graph = Graphical synthesis; Bold and cursive references = duplicate studies; *M* = mean; ns = Ns; MSM = Men who have sex with men; ASA = ASA; SIPV = Sexual Intimate Partner Violence; CSA = Child Sexual Abuse; I = Individal changes; IP = Interpersonal changes; + = Positive individual and interpersonal changes; C = Chronosystem factors; I = Individual level factors; A = Assault-related factors; MM = Micro-/Mesosystem factors; E = Exosystem factors; Ma = Macrosystem factors; (Ma) = Macrosystem factors are mentioned but no empirical studies are included.
