# Supplementary material for: An ecological approach to understanding the impact of sexual violence: a systematic meta-review
Source: Front Psychol. 2023 May 24;14:1032408. doi: 10.3389/fpsyg.2023.1032408 (PMC10244654; doi:10.3389/fpsyg.2023.1032408)
Supplement: Supplementary file 2 [file Table_2.pdf]

APPENDIX B. Covered changes

	Psychological changes	Social changes
**Alessi et al.(2021)**	Sexual violence is associated with substance abuse and post-traumatic stress	
**Alvarez-Segura et al. (2014)**	Survivors of SIPV showed a higher likelihood of reporting antenatal depressive symptoms compared to controls	
**Ba et al. (2017)**	24.6-76% of survivors experience PTSD related symptoms, 9-76% depression symptoms, 7-75% anxiety disorders, 3-50% alcohol and substance abuse, 15-39% suicidal ideation, 4-37% suicide attempts, 19% somatic complaints, 76% permanent headache, 25-57% sexual dysfunction and 13-18% engage in more sexual risk behavior	9-62% of survivors experience social dysfunction, 26% of survivors experience difficulties in the relationship with partner and spousal abandonment
**Bergmann et al. (2015)**	Survivors report decreased condom use and condom use negotiation than non-survivors	
**Beydoun et al. (2012)**	A greater likelihood of depression in survivors compared to non-survivors (RR = 2.45; 2.5 < OR < 4.41; 1 OR ns)	
**Bows (2018)**	Survivors report a low self-esteem, feelings of hopelessness, nightmares, flashbacks, sleeping problems, anxiety and fears about leaving their home/staying at home	
**Buller et al. (2014)**	No association of sexual violence and unprotected anal sex (OR ns)	
**Bundock et al. (2013)**	0%-15% of eating disorder patients has experienced SIPV	
**Callan et al. (2021)**	Sexual violence is associated with an increased risk of unprotected anal intercourse	
**Campbell et al. (2000)**	Survivors show greater levels of depression, a less positive self-image, a greater risk of becoming a victim of homicide as a consequence of intimate partner violence in comparison to non-survivors<br>31.9% of survivors report fear of STD's, 86% decreased condom use, 42.8% decreased contraceptive use<br>Survivors report feelings of powerlessness regarding sexual self-care<br>Survivors have an increased likelihood of gynecological problems including decreased sexual desire (OR = 2.65)	
**Chen et al. (2010)**	A greater likelihood of depression (OR = 8.87), anxiety (OR = 7.21) and suicide attempts (OR = 10.91) in survivors compared to non-survivors	
**Chmielowska et al. (2017)**	A greater likelihood of PTSD (OR = 4.57) and affective disorders (OR = 4.2) but not for anxiety (OR = ns) in survivors compared to non-(SIPV) survivors	
**Classen et al. (2005)**	A greater likelihood of depression, anxiety and revictimization in survivors compared to non-survivors	
**Coker (2007)**	A greater likelihood gynecological problems including problems with sexual desire and sexual pleasure (OR = 2.7)	
**Cook et al. (2011)**	More PTSD arousal and avoidance symptoms among survivors in comparison to non-survivors	
**Dame et al. (2020)**	Sexual violence is associated with depression, anxiety, post-traumatic stress disorder, fear, suicide, lack of sex drive, compulsive eating and substance use	Sexual violence is associated with social isolation

APPENDIX B. Covered changes (continued)

	Psychological changes	Social changes
**Davis et al. (2023)**	Survivors report feeling less confidence to refuse unwanted sexual advances, feeling less in control of themselves as a sexual being, feeling guilty about having sex with the perpetrator, feeling disrespected, exploited, upset, fearful, angry	
**de Souza Mezzavilla et al. (2018)**		A greater likelihood of an early start of breastfeeding (OR = 2.38 but only significant in Zambia) and early termination of exclusively breastfeeding (1.68 < OR< 11.11 for Ghana, Liberia and Bangladesh, OR = .40 for Zambia) in survivors compared to non-survivors
**Diez-Canseco et al. (2022)**	Combined prevalence of 26% (prevalence rates range from 5.1% to 67.9%) of survivors of sexual harassment at the workplace who experienced depression, survivors who have experienced sexual harassment at the workplace have 2.69 higher odds (odds ranged between 1.92 to 4.12) of depression compared to employees without harassment experience, having experienced sexual harassment at the workplace is associated with higher depression scores.	
**Dillon et al. (2013)**	Survivors of SIPV show a higher odd of depression (OR = 42.60) than survivors of physical (OR = 10.28) or emotional (OR = 5.83) abuse. SIPV survivors report more suicidal ideation, suicide attempts and lower mental health compared to controls. SIPV and physical abuse are associated with increased suicidal ideation (however, the latter is inconsistent). SIPV survivors report having decreased ability for sexual decision-making and negotiating condom use.	SIPV survivors report lower social functioning compared to controls
**Dworkin (2020)**	Sexual violence is associated with anxiety (OR = 3.05-3.20), depression (OR = 2.53-3.25), bipolar disorder (OR = 3.85), eating disorder (OR = 1.98), post-traumatic stress disorder (OR = 3.32-5.59) and substance use disorder (OR = 1.74-2.16) but not with obsessive-compulsive disorder (OR ns)	
**Dworkin et al. (2017)**	A greater likelihood of psychopathology (g = .61) among which suicidality (g = .74) trauma- and stressor-related conditions (g = .71), obsessive-compulsive conditions (g = .71), bipolar conditions (g = .66), depression (g= .60), anxiety (g = .53), disordered eating (g = .39), and substance abuse/dependence (g = .37)	
**Dworkin et al. (2021)**	Up to 81.42% of sexual violence survivors suffers from PTSD following the assault	
**Elderton et al. (2017)**	18-80% of survivors experience post-traumatic growth among which lifestyle changes, material gain, enhanced self-efficacy, spirituality as well as changes in beliefs about the world, strength and recovery process from victim to survivor, a change in power (themes from retrieved from quantitative and qualitative analyses)	18-80% of survivors experience post-traumatic growth among which family closeness, community closeness, faith in people and compassion/empathy.
**Feldner et al. (2007)**	Higher odds of ever smoking (OR = 3.52), currently smoking (OR = 3.4) and nicotine dependence (OR = 5.10) among sexual violence survivors compared to other trauma types, survivors of SIPV have higher odds of smoking (OR = 2.07) compared to other types of IPV	
**Finneran et al. (2012)**	More substance use/abuse (1 study with significant results, 4 studies with insignificant results, more alcohol	

APPENDIX B. Covered changes (continued)

	Psychological changes	Social changes
	l use (1 study with insignificant results), more depression symptoms (1 study with significant results, 4 studies with insignificant results), lower self-esteem (1 study with significant results), increased mental health problems (1 study with significant results), increased unprotected insertive and receptive anal intercourse (4 studies with significant results, 3 studies with insignificant results), a decreased HIV testing history (1 study with significant results, 1 study with insignificant results), a higher likelihood of trading sex (1 study with significant results, 1 study with insignificant results), a higher number of partners (1 study with significant results, 1 study with insignificant results) and being less able to talk with partner about condoms (1 study with significant results) than non-survivors	
**Forkus et al. (2021)**	Sexual violence survivors show a higher odds to report more legal problems/criminal justice involvement, disordered eating (only among women), depression, PTSD, substance use (inconsistent), suicidal ideation and behavior (inconsistent), suicide attempts (inconsistent), increased number of sexual partners, trading sex than non-survivors. No association was found with aggressive behavior.	
**Gallegos et al. (2021)**	All survivors of military sexual trauma reported sleep disturbance	
**Gielen et al. (2007)**	More high-risk sexual behavior, and a greater likelihood of having multiple sex partners in survivors compared to non-survivors	
**Godier-McBard et al. (2020)**	Sexual violence survivors report mood disturbances, sexual problems, shame, anger, being unsatisfied with their jobs, wanting to leave the military	Sexual violence survivors report difficulties with peer relationships
**Goodman et al. (1997)**	21-76% of patients with a severe mental illness (among which personality disorder, affective disorder, schizophrenia, substance use) experienced adult sexual abuse	
**Guggisberg et al. (2021)**	Post-traumatic growth regarding self: learning to connect with themselves, searching for meaning, taking control, becoming aware of emotions and thoughts, a positive future orientation, hope, positive self-perceptions, meaning making	Post-traumatic growth regarding others: feeling a sense of belonging by engaging in relationships with others, practicing yoga and spirituality in groups. Individual growth and social growth reinforce each other.
**Halstead et al. (2017)**		Survivors may experience interference with relationships, increased parental restrictions of survivors' parents
**Heerde et al. (2016)**	A greater likelihood of drug abuse (OR = 1.75), marihuana use (r = .26), and more alcohol (ab)use (one study shows a correlation of r = .31; another study shows OR = ns) in survivors compared to non-survivors	
**Kahsay et al. (2020)**	50.7-94.7% reports psychological symptoms, 8.2-67.9% depression, 11.4-80% fear/anxiety, 37.1-44% anger, 30.4% shame, 8.2-17.3% helplessness, 67.9% disappointment, 6.2% guilt/self-blame, 1.3-15% fatigue/feeling exhausted, 40.3% 57.5% a headache, 24.2%-56.7% difficulties sleeping, 48.3% nightmares, 8.6-35% loss of appetite, 54% sadness, 25% loss of self-confidence, 35% crying for no reason, 19% ferociousness, 48% bitterness,	22% of survivors reports social isolation, 19% trouble in emotional relationships, 36.8% negative social and family relationships
**Klein et al. (2021)**	Sexual violence is associated with an increased risk of abusing alcohol, experiencing psychological distress	
	Sexual violence is associated with post-traumatic growth, depression, anger, low life satisfaction and PTSD, lower academic satisfaction, disordered eating	

APPENDIX B. Covered changes (continued)

	Psychological changes	Social changes
**Klein et al. (2022)**	Sexual violence is associated with suicide attempts, PTSD symptoms, depressive symptoms but not with being a smoker	
**Kouvelis et al. (2021)**	Sexual violence is associated with greater self-perception impairments than not having experienced sexual violence, sexual violence is associated with greater self-perception impairments compared to other trauma-types	
**Kouyoumdjian et al. (2013)**	Survivors report a low self-worth, negative changes regarding sexuality (e.g., increased and decreased sexual frequency), fear of rejecting men among survivors	
**Langdon et al. (2017)**	Sexual violence is associated with alcohol use	
**Larijani et al. (2015)**	No association of dental fear with adult sexual violence, but there was an association with adult sexual and physical violence combined	
**Lim et al. (2022)**	Inconsistent results regarding the association of sexual violence with life satisfaction and mental health, depression and suicide attempts, sexual violence is associated with more risky sexual behavior, increased substance use, PTSD symptoms	Sexual violence is associated with lower perceived control and power within an intimate relationship
**Lombardi et al. (2023)**	Inconsistent results regarding prenatal depression: 3 studies found significant results (OR = 1.60-3.43) whereas 1 study did not find a significant effect (OR ns). Meta-analytical results also show no significant association (OR ns).	
**Madowitz et al. (2015)**	53% of survivors suffers from an eating disorder; 24.5% of patients with an eating disorder experienced sexual violence (according to one study this association is mediated by PTSD and depression), 13.7% of patients with anorexia have PTSD of which 35% was due to sexual violence, 85% of survivors reports aversion to sexual activity	
**Maman et al. (2000)**	A greater likelihood of HIV risk behavior (26-32% of survivors vs. 13-22% of non-survivors, significant difference), engaging in exchange sex, being less acquainted with sex partner and being afraid of negotiating condom use than non-survivors<br>A lower likelihood of condom use during the last sexual encounter than non-survivors.<br>Survivors are 6 times more likely to have had sex >10 times in the last three months, 3 times more likely to have never used condoms during the last three months and 11 times more likely to have a physically abusive partner than non-survivors	
**Mauritz et al. (2013)**	29-71% of the respondents with severe mental illness has experienced adult sexual violence, 37% of the respondents with bipolar disorder has experienced adult sexual violence, 8-37% of the respondents with depression has experienced adult sexual violence, 39-87% of the respondents with borderline personality disorder has experienced adult sexual violence, 6-48% of the respondents with schizophrenic spectrum disorder has experienced adult sexual violence	
**Mazza et al. (2021)**	Sexual violence is associated with perinatal depression and PTSD	
**Messman-Moore et al. (2003)**	Survivors report more alcohol use during dating, a higher number of sex partners, a higher number of consensual sexual experiences and a higher likelihood of sexual revictimization compared to non-survivors	ASA survivors and revictimized survivors show a higher likelihood of having multiple partners and brief relationships compared to child sexual abuse survivors or non-survivors, adolescent sexual violence is associated with interpersonal problems

APPENDIX B. Covered changes (continued)

	Psychological changes	Social changes
**Meyer, et al. (2011)**	Survivors report more substance abuse, a greater number of sex partners, more unprotected sex, less consistent condom use, less sexual assertiveness and a greater likelihood of experiencing intimate partner violence than non-survivors	
**Molstad et al. (2023)**	Survivors report lower GPA scores than controls (inconsistent), sexual violence is associated with academic impact (r=.12; OR = 2.33) and academic disengagement (r = .06), increased college stress and lower academic efficacy	
**Nicholas et al. (2022)**	Male survivors show a 3.6 times likelihood to report poor mental health including suicidal ideation, depression, anxiety, non-suicidal self-injury	
**Normann et al. (2020)**		Survivors of SIPV show a lower likelihood of initiating breastfeeding (OR = .52) than controls
**Paras et al. (2009)**	A greater likelihood of suffering psychogenic seizures (OR = 2.72) and chronic pelvic pain (OR = 3.03) in survivors compared to non-survivors<br>Fibromyalgia and headache are not associated with sexual violence (OR = ns)	
**Paulson (2020)**	Having experienced lifetime and recent SIPV is associated with a higher likelihood of postnatal depression (r = .37; OR = 1.84-8.23; however, inconsistent) and antenatal depression (OR = 14.3) compared to non-exposed women	
**Pebole et al. (2021)**	Weight trainers have a higher likelihood of having experienced sexual violence than regular runners, survivors of sexual violence report a higher likelihood of being sedentary, survivors of sexual violence show no higher likelihood of enrolling in a self-defense class however, they do report the assault as the reason for their enrollment.	
**Peterson et al. (2011)**	58-85% of survivors experience PTSD symptoms (flashbacks, dissociative symptoms, avoidance), 36% emotional distancing, 20-32% depression and other mood disorders, 23% anger, 23-50% fear and anxiety and taking precautions, 8% self-harm, 28-69% suicidal ideation and attempts, 32% lower self-worth and self-respect, 35-48% substance abuse, 12-50% sexual dysfunction, 26% conflicted sexual orientation, 19-33% decreased academic functioning<br>Survivors report difficulties with masculinity and sex role reputation, disordered eating, borderline personality disorder, distress, unprotected intercourse, exchange sex and risk-resistant behavior.	Survivors may experience problems with interpersonal functioning: 18-20% reports decreased involvement in social activities, 66-75% feelings of distrust of others 63-71% nervousness around people, 68-65% discomfort being physically close to others and 81-90% social withdrawal and social changes
**Pulverman et al. (2021)**	Survivors of sexual violence show a higher likelihood of sexual dysfunctions and lower likelihood of reporting a decreased sexual satisfaction than non-survivors and survivors of other trauma types, survivors of sexual violence report more sexual problems than the norm, 74.4% of survivors reports at least one sexual dysfunction	
**Pulverman et al. (2019)**	Higher number of sexual difficulties compared to the norm in military sexual violence survivors, no difference in sexual functioning between military sexual violence survivors and military controls, military survivors do report a lower sexual satisfaction than military controls; 74.4% of military survivors report sexual dysfunction; survivors are more likely to report depression, anxiety, abuse and sleep difficulties	

	Psychological changes	Social changes
**Rani et al. (2022)**	Survivors report fear, loss of control, sense of betrayal, feelings of guilt, powerlessness, anger, helplessness, developing a negative attitude towards men in general, developing an aversion towards sex, low self-esteem, self-blame and poor body image	Survivors report isolation
**Ribeiro et al. (2009)**	A greater likelihood of suffering from anxiety and depression symptoms (OR = 9.7), common mental disorders (OR = 4.4 ; however one study showed an insignificant OR) than non-survivors. For suicidal ideation the OR was insignificant.	
**Romans et al. (2008)**	87% of patients with IBS experienced sexual violence vs. 14.1% of healthy women (1.82 <OR <1.86), 23% of IBS patients experienced sexual violence vs 8% of physical gastro-intestinal disease patients, 30% of functional gastro-intestinal disorder patients has experienced sexual violence vs 5% of organic gastro-intestinal disorder patients, this difference was significant).<br>One study shows no difference between IBS patients (28%), chronic idiopathic constipation patients (34%), Chron's disease patients (25.5%) and healthy women (22.6%) regarding the prevalence of sexual violence. In addition, no difference was found between Fibromyalgia/ chronic fatigue syndrome patients (0%) and controls (0%) with regard to the prevalence of having experienced sexual violence.	
**Salim et al. (2022)**	Bisexual female survivors have a 37% increase in the odds for smoking compared to bisexual female controls in addition in bisexual women, there is an association between sexual violence and increased substance use	
**Scoglio et al. (2021)**	Childhood sexual violence predicts adolescent sexual violence which in turn predicts adult sexual violence	
**Seth et al. (2013)**	A decreased likelihood of condom use, having multiple sex partners, having 3 or more sex partners in the past 3 months, having sex while being intoxicated, having sex while partner is intoxicated and ever being pregnant than non-survivors<br>A lower likelihood of no condom use at last sexual encounter than non-survivors<br>A higher number of sex partners, more inconsistent condom use, more high-risk sexual behavior than non-survivors	
**Shamblaw et al. (2019)**	ASA is associated with prenatal depression ($.18 < r < .39$).	
**Sparrow et al. (2017)**	Significant higher PTSD scores for survivors (41.7) vs. non-survivors (29.7)<br>More problem drinking among survivors vs. non-survivors (no scores reported)<br>No difference in depression scores between survivors (19.5) and non-survivors (14.5)	
**Spencer et al. (2023)**	Having experienced sexual violence is associated with increased risk of revictimization, hopelessness, suicidal ideation, binge drinking, trauma symptoms, anxiety symptoms, disordered eating, risky sexual behavior, depressive symptoms, number of sexual partners, substance use, alcohol use, psychological distress, tobacco use and decreased sexual assertiveness	Sexual violence is associated with experiencing partner violence and interpersonal problems
**Steine et al. (2012)**	10% of survivors reports suffering from sleep disturbance (70% of survivors), 100% reports sleeping problems and 95% reports nightmares and flashbacks.	

APPENDIX B. Covered changes (continued)

	Psychological changes	Social changes
**Stewart et al. (2019)**	The majority of included studies show how survivors report an increase in alcohol use following the assault, some show no change. Survivors report an increase/decrease (inconsistent results) in sexual activity following the assault. Eating behavior decreases and after a while increases. Survivors report dressing differently and report an increase in drug use and smoking.<br><br>Survivors report a decrease in school/work attendance and leisure occupations	Survivors report a decrease in dating
**Stockman et al. (2013)**	A higher likelihood of >5 partners (OR = 1.8), casual partners (OR = 2.2) and exchange sex (OR = 1.8), unprotected sex (OR = 13.4), inconsistent condom use (OR = 3.31) and injection drug use (injection drug users are more likely to experience sexual violence, OR = 4.5) than non-survivors.<br>Sexual violence was associated with a lower condom use frequency, a lower sexual assertiveness regarding condom use and unprotected sex (through a lower sexual assertiveness and depression symptoms) and sexual and drug risk behavior	
**Trevillion et al. (2012)**	44.8% of survivors is be diagnosed with PTSD<br>No significant difference was found in the prevalence of depression and personality disorders among survivors and non-survivors.	
**Ullman (2004)**	Survivors report more (past year) suicidal ideation, suicide attempts and feelings of hopelessness<br>An increased risk of a lifetime suicide attempt among survivors compared to non-survivors However, the association between sexual violence and suicidal behavior is not found in all studies	
**Ulloa et al. (2016)**	20-50% of survivors report little to great change in the positive direction, 30% of survivors report become involved in social and political action/became an advocate<br><br>Survivors may report post-traumatic growth, increased spirituality, increased sense of well-being, PTSD, depression, distress, appreciation of life, spiritual change, personal strength, stronger sense of self and new possibilities	Survivors may report changes such as an increased relationship with their mother, relationship with others, more empathy towards others
**van Berlo et al. (2000)**	A 2.5 times higher likelihood to experience sexual dysfunctions (i.e., arousal dysfunction, desire dysfunction, vaginismus, dyspareunia, anorgasmia, organismic disorder, fear of sex, aversion towards sex, sexual noncommunication, sexual feelings, feelings of sensuality), 30% of survivors report sexual problems, 22-33% decreased sexual satisfaction, 32% decreased frequency of having sex, 29-38% avoiding sex for at least 6 months, 50% flashbacks during sex, 14-34% finding sensual contact less pleasurable, 30% having PTSD symptoms and 4% depression symptoms.<br>Survivors report a decrease in orgasms<br>Survivors are less sensual compared to controls<br>Some studies found no differences between victimized and non-victimized respondents regarding the frequency of oral sex, sexual intercourse, anal intercourse, masturbation, orgasms, sexual avoidance, sexual noncommunication, vaginismus, affectional experiences and autoerotic experiences	
**Vitek et al. (2021)**		Having experienced sexual violence is associated with experiencing physical violence in intimate relationships, decreased relationship satisfaction, poorer communication

APPENDIX B. Covered changes (continued)

	Psychological changes	Social changes
		(however, inconsistent), poorer emotional intimacy (but not sexual intimacy). No association is found with hostility
**Wadsworth, et al. (2013)**	A higher likelihood of experiencing depression and anxiety symptoms (b = 1.37; 2.96 < OR < 5.32), PTSD symptoms (3.05 < OR < 7.15; R = .40) and somatic symptoms (OR = 3.65) compared to non-survivors.<br>One study stated that recent sexual violence is not associated with anxiety symptoms and another study stated that there was no association with suicidal ideation.<br>Sexual violence is associated with more substance use, illicit drug use, alcohol abuse, inconsistent condom use, unprotected vaginal, anal or oral sex, number of partners, practicing monogamy and trading sex compared to non-survivors.	
**Walker et al. (2017)**	A mean prevalence rate of revictimization of 47% following sexual violence among adolescents	
**Wilness et al. (2007)**	Sexual violence is associated with decreased mental health (r = -.27) and life satisfaction (r= .12) and increased PTSD symptoms (r = .25)<br>Sexual violence is associated with decreased co-worker satisfaction (r = -.32), supervisor satisfaction (r = -.29), work satisfaction (r = -.24), global job satisfaction (r = -.25), organizational commitment (r = -.25), workgroup productivity (r = -.22) and increased job withdrawal (r = .16)	
**Zarchev et al. (2021)**	5.3% of men with a mental illness has experienced past-year sexual violence whereas 14.1% of men with a mental illness has experienced adulthood sexual violence	

Note. PTSD = Post-traumatic stress syndrome, STD = Sexually transmitted disease
