# Supplementary material for: An ecological approach to understanding the impact of sexual violence: a systematic meta-review
Source: Front Psychol. 2023 May 24;14:1032408. doi: 10.3389/fpsyg.2023.1032408 (PMC10244654; doi:10.3389/fpsyg.2023.1032408)
Supplement: Supplementary file 3 [file Table_3.pdf]

APPENDIX C. Covered risk and protective factors per level and their associations with outcomes and/or other levels

	Chronosystem	Individual	Assault	Micro-/Mesosystem	Exosystem	Macro system
**Alessi et al.(2021)**					Feeling pressure to reveal details about the assault is associated with chronic stress and anxiety	
**Ba et al. (2017)**		- A trend of females being more likely to report PTSD (24.6-76% vs 36-56%), depression (9-76% vs 33-48%) and anxiety symptoms (7-75% vs 14%), more suicide attempts (10-37% vs 4-23%), than males but it depends on the individual study<br>- A trend of males being more likely to report substance use (3-50% vs 3-18%) and sexual risk behavior (18 vs 13%) than females<br>- A trend of no difference regarding suicidal ideation between males and females (15-39% vs 18-37%)				
**Bird et al. (2021)**		- PTSD is associated with decreased sexual functioning<br>- PTSD among MST survivors is associated with increased sexual distress among European-American women but not among African-American women				
**Bows (2018)**		Both older male and female survivors report low self-esteem and hopelessness				
**Campbell et al. (2000)**		Feelings of powerlessness (in combination with fear of physical and emotional abuse) may play a role in more high-risk sex behavior (theme resulted from qualitative analysis)				

APPENDIX C. Covered risk and protective factors per level and their associations with outcomes and/or other levels (continued)

	Chronosystem	Individual	Assault	Micro-/Mesosystem	Exosystem	Macro system
**Classen et al. (2005)**	- The more recent or severe the index sexual violence, the higher the odds of becoming revictimized<br>- Having a history of CSA or ASA before the index sexual violence (vs. one-time ASA only) is associated with more distress and depression (although inconsistent results), more suicide attempts, a higher likelihood of receiving a lifetime PTSD diagnosis (although inconsistent results), more anxiety, more dissociation, more alcohol use, a higher likelihood of suffering from a dissociative disorder, more feelings of powerlessness, more difficulties processing sexual trauma related stimuli, more negative expectations of others, more rigidity in interpersonal schemas, more problems being sociable or intimate, more controlling, more substance use and a higher risk of revictimization		- Sexual violence by a peer is not associated with a higher risk of revictimization.<br>- The use of force during the index sexual violence increases the likelihood of becoming revictimized.<br>- The severity of the abuse (and not revictimization) predicts PTSD severity			
**Cook et al. (2011)**	- More recent sexual violence is associated with more PTSD symptomatology<br>- More time since the sexual violence is associated with decreased depression levels	- Both older and younger women show a drop in depressive symptoms 4 months after the sexual violence<br>- 12 months after the sexual violence an older age was associated with more depression				

APPENDIX C. Covered risk and protective factors per level and their associations with outcomes and/or other levels (continued)

	Chronosystem	Individual	Assault	Micro-/Mesosystem	Exosystem	Macro system
**Diez-Canseco et al. (2022)**	- Having experienced more than one instance of sexual harassment at the workplace is associated with higher odds of depression (4.12) vs. the odds when having experienced one instance of harassment (1.44)					
**Dworkin et al. (2017)**	The effect of ASA on psychopathology does not differ as a function from years since the assault (effect size not significant)	Age, gender and ethnicity are not associated with psychopathology (effect size not significant)	- Completed vs attempted rape (Δg =-.10), being injured (b = .18) and the presence of a weapon (b = .21) are positively associated with psychopathology.<br>- Non-penetrative sexual violence and the relationship with the perpetrator are not associated with less psychopathology			
**Dworkin et al. (2019)**				- Positive social reactions as well as negative social reactions were positively associated with psychopathology (r = .05; r = .22) whereas perceived positive social reactions were negatively associated with psychopathology (r = -.13).<br>- However, prospectively, positive social reactions were not associated with psychopathology; the negative association between perceived positive social reactions and psychopathology remained (however, not specifically tested for adult sexual violence).	Whether the same pattern with perceived social reactions applies to formal support providers as well remains unclear	

APPENDIX C. Covered risk and protective factors per level and their associations with outcomes and/or other levels (continued)

	Chronosystem	Individual	Assault	Micro-/Mesosystem	Exosystem	Macro system
**Dworkin et al. (2021)**	The decline of post-traumatic stress disorder prevalence and symptom severity occurs faster in the first three months following the assault compared to the period between three to twelve months post-assault (81.42% prevalence to 53.60% prevalence from one week - three months vs. 53.60% to 41.49% prevalence from three months to twelve months)	An older age is associated with a steeper decline of PTSD prevalence and symptom severity in the first three months following the assault				
**Elderton et al. (2017)**	- Sexual revictimization is negatively associated with post-traumatic growth<br>- Post-traumatic growth increases over time (20-80% of survivors reports PTG 2 weeks post-assault, 37-81% 2 months post-assault, 37-72% 6 months post-assault and 39-76% 1 year post-assault)	- Hardiness, acceptance coping, religious coping, approach coping, perceived control over recovery and non-Caucasian and African-American ethnicity are positively associated with post-traumatic growth<br>- Avoidance coping is negatively associated with post-traumatic growth	The severity of sexual violence is unrelated to level of post-traumatic growth	Informal social support is associated with more post-traumatic growth	Formal social support is associated with more post-traumatic growth	
**Feldner et al. (2007)**			Severity of sexual coercion is positively related to nicotine dependence			
**Forkus et al. (2021)**		Sexual violence is only associated with eating disorders among women				
**Galatzer-Levy et al. (2018)**	Sexual violence survivors do not experience delayed onset PTSD symptoms					
**Gielen et al. (2007)**		Avoidant coping is associated with high-risk sex and multiple partners among females and not among males		Sexual violence is associated with high risk sex and multiple partners in singles, but not in survivors with partner		
**Godier-McBard et al. (2020)**		Male survivors show higher scores on psychopathic traits,	Male survivors of same-sex sexual violence experience			

APPENDIX C. Covered risk and protective factors per level and their associations with outcomes and/or other levels (continued)

	Chronosystem	Individual	Assault	Micro-/Mesosystem	Exosystem	Macro system
		paranoia, schizophrenia and hypomania than female survivors	more pervasive and severe consequences than male survivors of non-same-sex sexual violence, hyper-heterosexuality and conflicting sexual orientation reported only among male survivors			
**Gong et al. (2019)**		- Pre-existing alcohol use problems are associated with less PTSD symptoms immediately following the assault but more symptoms one month to three months later<br>- Characterological and behavioral self-blame is associated with increased PTSD	- Inconsistent evidence regarding the role of acute intoxication on trauma symptoms: cross-sectional studies found a greater PTSD symptomatology among intoxicated survivors than sober survivors whereas other studies found that forcible rape is associated with higher PTSD symptoms compared to incapacitated rape<br>- Other cross-sectional studies did not find an association<br>- Longitudinal studies found less intrusive symptoms but a slower recovery in intoxicated survivors than in sober survivors, other studies suggest no significant difference<br>- Survivors who were intoxicated during the assault report more alcohol use following the assault than survivors who were sober<br>- The association from intoxication to PTSD is mediated by characterological self-blame and negative social reactions.	Negative social reactions are associated with increased PTSD symptoms.		

APPENDIX C. Covered risk and protective factors per level and their associations with outcomes and/or other levels (continued)

	Chronosystem	Individual	Assault	Micro-/Mesosystem	Exosystem	Macro system
**Halstead et al. (2017)**				- Increases in perceived and actual social support may lead to positive self-appraisals<br>- Unsupportive reactions are associated with negative self-cognitions, PTSD-symptoms, depression, anxiety and lower-self-esteem.<br>- Nondisclosure is associated with less post-traumatic growth and an increased chance of revictimization		
**Hellman (2014)**	Additional traumas are associated with a greater likelihood of suicidal ideation and attempts	- Self-blame is associated with greater suicidal ideations.<br>- General self-blame, characterological self-blame, behavioral self-blame and anticipatory blame re associated with depression, PTSD, social withdrawal, psychological distress, revictimization, less post-traumatic growth and suicidal ideation<br>- Behavioral self-blame mediates the association between unsupportive reactions and problem drinking<br>- Religious coping is associated with a lower well-being<br>- Avoidance coping, being younger, belonging to a minority group and identifying as bisexual is associated with more suicidal ideation		- Disclosure is associated with more suicide attempts<br>- Positive social reactions are associated with more disclosure and better relationships<br>- Unsupportive social reactions are associated with PTSD		

APPENDIX C. Covered risk and protective factors per level and their associations with outcomes and/or other levels (continued)

	Chronosystem	Individual	Assault	Micro-/Mesosystem	Exosystem	Macro system
		- Avoidance coping is associated with PTSD symptoms<br>- Greater control over recovery is related to fewer suicide attempts				
		- Increased perceptions of control over recovery is associated with decreased depression, anxiety and PTSD symptoms and decreases in suicidal ideation				
**Kennedy et al. (2018)**	Revictimization is related to greater internalized stigma than adult sexual violence only	Self-blame is associated with PTSD through maladaptive coping as a mediator		- Unsupportive victim-blaming reactions from informal support providers are associated with survivors' reduced self-esteem, problem drinking, self-blame, maladaptive coping, hostility, reduced sexual assertiveness, revictimization, paranoia, increased rates of PTSD, depression (however, inconsistent), and health problems<br>- Negative reactions from intimate partners are perceived as most hurtful	Negative reactions from formal systems are associated with PTSD and psychological distress	
**Klein et al. (2021)**		Male survivors' show more problematic drinking than female survivors, psychological distress is associated with disengagement of the academic environment and lower academic performance, ethnic minority students experience more shame, depression and clinical				

	Chronosystem	Individual	Assault	Micro-/Mesosystem	Exosystem	Macro system
		symptoms compared to white students.				
**Klein et al. (2022)**		- Sexual minority survivors report lower mental health and life satisfaction more depressive symptoms, anxiety, suicidal ideation, self-harm disordered eating, alcohol use, body shame, and anticipated more negative reactions than non-minority survivors<br>- No difference is found regarding suicide attempts				
**Knight et al. (2022)**		Ability to adapt to life changes, positive thinking, intelligence, self-esteem, self-efficacy, coping flexibility and spirituality are associated with greater resilience		Informal social network and support (from family and friends) is associated with greater resilience	Formal social network and support is associated with greater resilience	
**Kouvelis et al. (2021)**		PTSD and depression symptoms are associated with greater impairment in self-concept				
**Langdon et al. (2017)**		Alcohol use increases the likelihood of revictimization; Effect sizes of the relationship between military sexual trauma and substance use were stronger among female survivors than among male survivors				
**LoGiudice (2017)**	Giving birth may reactivate memories of the rape and may retraumatize survivors (qualitative study)					
**Messman-Moore et al. (2003)**	No difference between revictimized survivors and ASA only survivors regarding PTSD symptoms					

APPENDIX C. Covered risk and protective factors per level and their associations with outcomes and/or other levels (continued)

	Chronosystem	Individual	Assault	Micro-/Mesosystem	Exosystem	Macro system
**Molstad et al. (2023)**	More types of sexual violence is associated with lower GPA scores (r between -.22 and -.29)					
**Nicholas et al. (2022)**		Male survivors report more mental health difficulties than female or gender-minority survivors				
**Ozer et al. (2003)**	Having experienced previous sexual violence is associated with a greater likelihood of post-traumatic stress disorder (r = between .40 and .43)			Making use of an informal support network is associated with a decreased likelihood of post-traumatic stress disorder (r = -.42)		
**Pebole et al. (2021)**		Exercise improves survivors' mental and emotional health				
**Peterson et al. (2011)**		- Male survivors experience less changes and less severe changes than female survivors and consider the violence as moderately upsetting<br>- Higher levels of distress (anxiety, depression, intrusive experiences), psychological symptoms, sexual dysfunction, sexual concerns, concern about sex-role reputation, lifetime history of psychological disorders, psychiatric hospitalizations, suicidal ideation and suicide attempts in male than female victims.<br>- No significant differences between gay, lesbian and bisexual individuals regarding psychological distress, depression, and PTSD	- For male survivors, sexual violence by a female perpetrator is reported as less upsetting and is associated with less distress than sexual violence by male perpetrators<br>- Sexual violence by a male perpetrator is associated with more distrust in men<br>- Regardless of the perpetrator's sex, sexual confusion may be present after sexual violence among male survivors		Lack of treatment is associated with suicide attempts	
**Pulverman et al. (2021)**		- Survivors of sexual violence with a depression				

APPENDIX C. Covered risk and protective factors per level and their associations with outcomes and/or other levels (continued)

	Chronosystem	Individual	Assault	Micro-/Mesosystem	Exosystem	Macro system
		diagnosis show a higher likelihood of sexual dysfunctions than survivors without depression diagnosis<br>- Depression and PTSD are associated with increased sexual dysfunction<br>- Anhedonia PTSD symptoms and negative alterations in mood and cognitions mediate the relationship between sexual violence and sexual function/satisfaction<br>- Sexual satisfaction mediates the relationship between military sexual trauma and PTSD, depression and suicidality				
**Pulverman et al. (2019)**	Additional post-military sexual trauma is associated with orgasm dysfunction	- Sexual satisfaction mediates the relationship between military sexual trauma and PTSD, depression and suicidality<br>- Depression and PTSD are associated with sexual dysfunction<br>- PTSD and antidepressant use are associated with lower sexual desire<br>- Younger age and depression symptoms are associated with orgasm disorder and sexual pain, identifying as heterosexual is associated with sexual pain<br>- Mental health mediates the association of military sexual trauma and sexual dysfunction; survivors with depression diagnosis showed higher rates of sexual dysfunction than				

APPENDIX C. Covered risk and protective factors per level and their associations with outcomes and/or other levels (continued)

	Chronosystem	Individual	Assault	Micro-/Mesosystem	Exosystem	Macro system
		survivors without depression diagnosis				
**Rani et al. (2022)**	Revictimized survivors report having paranoid thoughts, post-trauma substance abuse, post-traumatic stress disorder, depression, anxiety, sexual dysfunction, insomnia, somatization, dissociation, and suicidal thoughts or attempts	Being hopeful, having a positive outlook on life, being resilient, having a sense of humor helps survivors to find meaning and to look for help.				
**Salim et al. (2022)**	Having experienced multiple instances of sexual violence is associated with more substance use than having experienced one instance of sexual violence	Bisexual survivors report more substance use, PTSD and depression than heterosexual survivors		- Bisexual survivors report more negative reactions than heterosexual survivors<br>- Negative reactions are associated to PTSD and substance use; the association between negative reactions and substance use is stronger for bisexual women than heterosexual women		
**Seth et al. (2013)**		A higher number of sexual partners among survivors labelled as Latinx, African American and White adolescents whereas a decreased likelihood of condom use is only found among Latino adolescents		Being single (vs. having a partner) is associated with more inconsistent condom use and a higher number of sex partners.		
**Sinko et al. (2022)**		A healing process consists of (1) processing trauma and reexamination: making meaning of what happened and understanding oneself, (2) Managing negative states: negative thoughts, emotions		A healing process consists of connecting with trusted others and building personal and professional support networks	A healing process consists of connecting with trusted others and building personal and professional support networks	

APPENDIX C. Covered risk and protective factors per level and their associations with outcomes and/or other levels (continued)

	Chronosystem	Individual	Assault	Micro-/Mesosystem	Exosystem	Macro system
		and posttraumatic symptoms and being able to cope with these, (3) rebuilding the self: recreating an identity, rebuilding self-esteem and self-worth, turning to spirituality and prioritizing their own well-being, (4) regaining hope and power: executing plans, making their own choices, being self-determined, finding a purpose and engaging in activities that give them purpose, building hope and reframing thoughts to create room for new possibilities in life				
**Sparrow et al. (2017)**			No significant mean difference in PTSD scores between non-intimate and intimate partner violence survivors (M = 42 vs M = 41).			
**Tolin et al. (2008)**		No significant difference between male and female regarding PTSD symptoms (OR insignificant)				
**Ullman (1999)**		- Avoidance coping strategies are associated with more psychological symptoms whereas approach coping is not associated or negatively associated with symptoms<br>- Avoidance coping is associated with less distress initially but with more distress at 6 to 12 months after the sexual violence.<br>- A high internal and low external locus of control is associated with adaptive coping<br>Having more control over		- Delayed disclosure is associated with poorer adjustment<br>- Having unsupportive social network members (vs. neutral or supportive network members) and unsupportive social reactions are associated with experiencing more psychological symptoms and a poorer self-rated recovery. This association would be mediated through avoidance coping<br>- The association of negative reactions with psychological adjustment	- Physicians were seen as the least supportive of all support providers rated, although their supportiveness was positively related to adjustment in contrast to supportiveness ratings of police and informal support sources	

APPENDIX C. Covered risk and protective factors per level and their associations with outcomes and/or other levels (continued)

	Chronosystem	Individual	Assault	Micro-/Mesosystem	Exosystem	Macro system
		the recovery is associated with positive life change<br>- Characterological and behavioral self-blame is related to a poorer psychological adjustment		did not differ across types of (in)formal support sources<br>- Some unsupportive reactions seem to be unrelated to the victim's perceived health<br>- Poor spousal support and unsupportive spousal behaviors are associated with experiencing more psychological symptoms, especially when this lack of support was unexpected<br>- Social support and supportive social reactions are associated with decreased depression symptoms, better recovery and better perceptions of physical health (however, only a small number of associations found between social reactions and outcomes, and inconsistencies are found in some studies: social support is not always associated with psychological adjustment)<br>- Supportive reactions from friends are more strongly related to better recovery than support or supportive reactions from other support providers. However, another study shows that friends were seen as most supportive, followed by family and boyfriends, and then police but none of these supportiveness ratings was significantly associated to psychological adjustment<br>- Closeness to important others was not associated to		

APPENDIX C. Covered risk and protective factors per level and their associations with outcomes and/or other levels (continued)

	Chronosystem	Individual	Assault	Micro-/Mesosystem	Exosystem	Macro system
				recovery initially, but family closeness was associated to better recovery 6 months following the sexual violence<br>- Unsupportive reactions are associated with problem drinking, especially among revictimized survivors.<br>- Living with family (vs. alone or with a spouse) is associated with less traumatization<br>- Marital status, quality of the victim's relationship with the spouse, family closeness soon after the assault is not associated with psychological symptoms		
**Ullman (2004)**	Sexual revictimization is associated with more suicidal ideation and attempts and an increased risk of a lifetime suicide attempt than ASA only	In one study the association of sexual violence with suicide ideation and attempts is only found among female survivors	Unwanted vaginal penetration following threat or physical force is associated with more suicidal behavior compared to no threat or force	Greater social support is associated with less suicide ideation and attempts in bivariate analyses (not in multivariate analyses)		
**Ulloa et al. (2016)**	Most post-traumatic growth occurs between 2 weeks and 2 months post-sexual violence	- Hardiness, hopefulness, an affective personality, being more religious, more perceived control over recovery, lower education, a younger age (however inconsistent: older age as well), being labelled as non-white, being religious and less neuroticism are associated with more post-traumatic growth<br>- Hardiness is also associated with less depressive symptoms	- No significant difference between sexual violence by a stranger or acquaintance regarding post-traumatic growth<br>- Greater sexual violence severity is suggested to be associated with more post-traumatic growth; however inconsistent results suggest a curvilinear relationship	- Positive reactions from family, friends are associated with more post-traumatic growth<br>- Being married is associated with less post-traumatic growth	- Positive reactions from support providers are associated with more post-traumatic growth	
**van Berlo et al. (2000)**	Sexual satisfaction, frequency of having sex, enjoying sex	Anger towards self, shame and guilt is associated with	Inconsistent results:	- Having a partner is associated with being		

APPENDIX C. Covered risk and protective factors per level and their associations with outcomes and/or other levels (continued)

	Chronosystem	Individual	Assault	Micro-/Mesosystem	Exosystem	Macro system
	and being orgasmic decreased after sexual violence, but increased or returned to pre-sexual violence levels when time since sexual violence increased (e.g. 30% survivors enjoyed sex 4 weeks post-assault, by 48 weeks 45% enjoyed sex; 43% of survivors were not sexually active 4 weeks post-assault, by 48 71% of survivors were sexually active).	fear of sex, lack of desire and aversion	- Having experienced attempted rape is associated with less sexual problems than having experienced completed rape (25 vs 50% experienced sexual problems). Of all respondents with sexual problems, 30% experienced complete rape vs 15% of the respondents without problems	sexually active after the sexual violence<br>- Having a partner and being sexually active is associated with less sexual problems<br>- Social support and satisfaction with the intimate relationship before the sexual violence are also associated with less sexual problems		
	However inconsistent results: fear of sex, sexual aversion, desire and arousal problems, and anorgasmia were present a year after the sexual violence and hardly decreased which made survivors distressed about the dysfunctions		- Liking the offender, not consuming alcohol, confidence-inducing strategies, physical violence, verbal threats, longer lasting sexual violence were associated with a higher likelihood to develop a sexual disorder<br>- Penetration during the sexual violence is associated with fear of sex<br>- Relationship to the perpetrator, penetration and degree of physical violence are not related to being sexually active<br>- The degree of physical violence is not associated with long-term sexual problems<br>- The identity of the perpetrator (acquaintance such as partner or date vs stranger sexual violence) is not associated with sexual			

APPENDIX C. Covered risk and protective factors per level and their associations with outcomes and/or other levels (continued)

	Chronosystem	Individual	Assault	Micro-/Mesosystem	Exosystem	Macro system
			satisfaction and sexual dysfunction<br>- A known perpetrator is associated with aversion of sex and lack of desire			
- **Wadsworth, et al. (2013)**	- Sexual revictimization is associated with PTSD (OR = 2.97) whereas ASA only was not<br>- Sexual revictimization is associated with more suicidal ideation than ASA only<br>- Revictimized survivors report higher stress than ASA only survivors (M = 2.8 vs M = 2.29)	Being labelled as White (vs. African American) is associated with more PTSD-symptoms	- Among African American survivors: current partner SIPV is associated with PTSD (b = .30), stress (R = .21) and dissociation (R = .32), non-partner sexual violence is associated with PTSD (b=.13) and past partner-SIPV is not associated with any outcome			
			- Among White survivors: current and past-SIPV is associated with PTSD, stress and dissociation whereas non-partner SV is not associated with any outcomes			
**Wright et al. (2022)**				Seeking informal help decreases the risk of future SIPV and improves health outcomes; negative reactions towards disclosure are associated with increased PTSD symptoms	Seeking formal help decreases the risk of future SIPV and improves health outcomes; negative reactions towards disclosure are associated with increased PTSD symptoms	

Note. SIPV = Sexual intimate partner violence, PTSD = Post-traumatic stress syndrome

APPENDIX C. Covered risk and protective factors per level and their associations with outcomes and/or other levels (continued)
